# Disturbed regulation of immunothrombosis in cerebral ischemia associated with SARS-CoV-2 infection

**DOI:** 10.3389/fimmu.2026.1662418

**Published:** 2026-01-29

**Authors:** Richard Plem, Nicole de Buhr, Rabea Imker, Silke Akhdar, Marita Meurer, Christine S. Falk, Johanna Ernst, Maria M. Gabriel, Jana Keil, Verena Kopfnagel, Thomas Illig, Karin Weissenborn, Sabine Blaschke, Isabel Bröhl, Christoph Römmele, NAPKON Investigators, Gerrit M. Grosse, Ramona Schuppner

**Affiliations:** 1 Department of Neurology, Hannover Medical School, Hannover, Germany; 2 Institute of Biochemistry, University of Veterinary Medicine Hannover, Hannover, Germany; 3 Research Center for Emerging Infections and Zoonoses (RIZ), University of Veterinary Medicine Hannover, Hannover, Germany; 4 Institute of Transplant Immunology, Hannover Medical School, Hannover, Germany; 5 Hannover Unified Biobank, Hannover Medical School, Hannover, Germany; 6 Emergency Department, University Medical Center Goettingen, Goettingen, Germany; 7 Goethe University Frankfurt, Faculty of Medicine, Institute for Digital Medicine and Clinical Data Sciences, Frankfurt, Germany; 8 Internal Medicine III – Gastroenterology and Infectious Diseases, University Hospital of Augsburg, Augsburg, Germany; 9 Department of Neurology and Stroke Center, University Hospital Basel, Basel, Switzerland

**Keywords:** acute ischemic stroke, immunothrombosis, neutrophil extracellular traps, SARS-CoV-2, transient ischemic attack

## Abstract

**Background:**

During the COVID-19 pandemic, it became evident that an infection with SARS-CoV-2 is associated with an increased predisposition for thrombembolic events. Recent studies suggest an excessive neutrophil extracellular trap (NET)-formation in response to SARS-CoV-2, which is considered a hallmark in immunothrombosis. A better understanding of the (dys)regulation of NET-formation in COVID-19 may provide the basis for new therapeutic strategies.

**Methods:**

We conducted a pilot study with a total of 84 patients in three groups matched in a 1:1:1 fashion: Group 1: patients with acute ischemic stroke (AIS) or transient ischemic attack (TIA) and SARS-CoV-2 infection, Group 2: patients with AIS and no SARS-CoV-2 infection and Group 3: patients with SARS-CoV-2 infection and no AIS or TIA. Venous blood samples were collected from all patients and subsequently analyzed for NET-specific markers, NET regulators (Deoxyribonuclease (DNase) activity) and a panel of cytokines.

**Results:**

Citrullinated histone3 (H3cit) and elastase levels were higher in groups with SARS-CoV-2 infection (Groups 1 and 3) compared to patients without ((group 1: H3cit = 2.9 (1.11–6.89) ng/mL, elastase = 312.1 (162–435.4) ng/mL and group 3: H3cit = 3.31 (2.03–7.97) ng/mL and elastase = 433.1 (281–783.8) ng/mL) vs. group 2: H3cit = 1.17 (0.61–2.15) ng/mL), elastase = 195.1 (91.99–386.9) ng/mL). No relevant differences were found regarding other measured NET-marker (myeloperoxidase, LL-37). DNase activity was lower in group 1 (6.12 (4.97–6.78) pmol/mL/min) compared to both other groups (group 2: (7.16 (5.88–7.85) pmol/mL/min) (p=0.018) and group 3 (7.19 (5.52–8.54) pmol/mL/min) (p=0.013).

**Conclusion:**

This pilot data suggest that a disturbed regulation of NETs in patients with SARS-CoV-2 infection may play a role in SARS-CoV-2 associated cerebral ischemia. These results highlight the importance of further investigating the role of NETs in immunothrombosis in the context of viral infections, to better understand its potential as a target for therapeutic strategies.

## Introduction

Severe acute respiratory syndrome coronavirus type 2 (SARS-CoV-2) infection comes along with higher risk for severe thrombotic diseases, including acute ischemic stroke, as pointed out by several studies at an epidemiological level (1, 2). It was revealed that thrombi found in the brain, heart, kidneys, and lungs of COVID-19 patients often contain neutrophil extracellular traps (NETs), indicating that NETs may play a crucial role in pathogenesis of thromboembolic complications during SARS-CoV-2 infection (3–5).

NETs are web-like structures primarily produced by neutrophils during an immune response (6). NETs consist of an extracellular DNA scaffold on which histones and antimicrobial peptides, as well as several other proteins, are bound (6, 7). These structures are expelled through a process known as NETosis and are capable of entrapping pathogens, preventing their further progression (6, 7).

Several viruses and bacteria, including the influenza virus and SARS-CoV-2 are known to increase circulating NET markers in blood (4, 5, 8, 9). Also, findings suggest that NETs promote the formation of thrombi through interaction and aggregation with von Willebrand factor (vWF) and platelets (10).

Other substances detected at elevated levels in the blood during SARS-CoV-2 infection include proinflammatory cytokines such as interleukin 6 (IL-6) and tumor necrosis factor alpha (TNF-α) (11, 12). These cytokines also exhibit prothrombotic effects by increasing the expression of activated tissue factor, thereby promoting the coagulation cascade and facilitating microthrombi formation in terms of a vicious cycle (11).

However, an important uncertainty persists regarding why some patients with SARS-CoV-2 infection develop thrombotic complications, while others do not. It is conceivable that patients with acute ischemic stroke or transient ischemic attack (TIA) during active SARS-CoV-2 infection exhibit altered endogenous NET and cytokine levels and regulation, leading to higher concentration of both NETs and cytokines, resulting in a state of systemic hypercoagulability. If differences in NET-formation and degradation and cytokine release are observed in patients with SARS-CoV-2 infection and acute ischemic stroke/TIA, this would indicate dysregulated activity in those pathways and suggest the potential for anti-NETosis or cytokine-modulating therapy to prevent or treat such complications in COVID-19 patients.

In this study, we therefore aimed to investigate whether NET-formation and/or reduced degradation and/or cytokine release, could be implicated in cerebral ischemia related to SARS-CoV-2 infection.

## Methods

To explore potential differences in NET markers and cytokine levels in patients who suffered an ischemic stroke or TIA while testing positive for SARS-CoV-2, we conducted a case-control study including one case group and two control groups, with a total of 84 patients. The control groups consisted of patients who either suffered an ischemic stroke/TIA or had a SARS-CoV-2 infection. All patients were matched in a 1:1:1 fashion based on demographic data, with patients with acute ischemic stroke without SARS-CoV-2 infection additionally matched for stroke severity, and SARS-CoV-2-positive controls matched for COVID-19 severity and the time interval between symptom onset and blood sampling. This study was conceived and conducted as a pilot investigation designed to explore feasibility, identify preliminary immunological patterns, and inform the design of future, larger studies. Given the exploratory nature of the pilot study, no formal sample size calculation was performed; all eligible patients within the designated recruitment period were included. We analyzed NET-specific markers (citrullinated histones (H3cit), elastase, myeloperoxidase (MPO) activity, LL-37) as well as NET-regulators (deoxyribonuclease (DNase) activity) and a panel of cytokines across all subgroups to assess their activity and levels to identify potential differences between these groups.

We followed the STROBE (Strengthening the Reporting of Observational Studies in Epidemiology) guidelines (13) to ensure comprehensive reporting of our study methodology and findings. The according checklist can be found in the **Supplementary Material**.

### Study cohorts and patient selection

**Figure 1:**
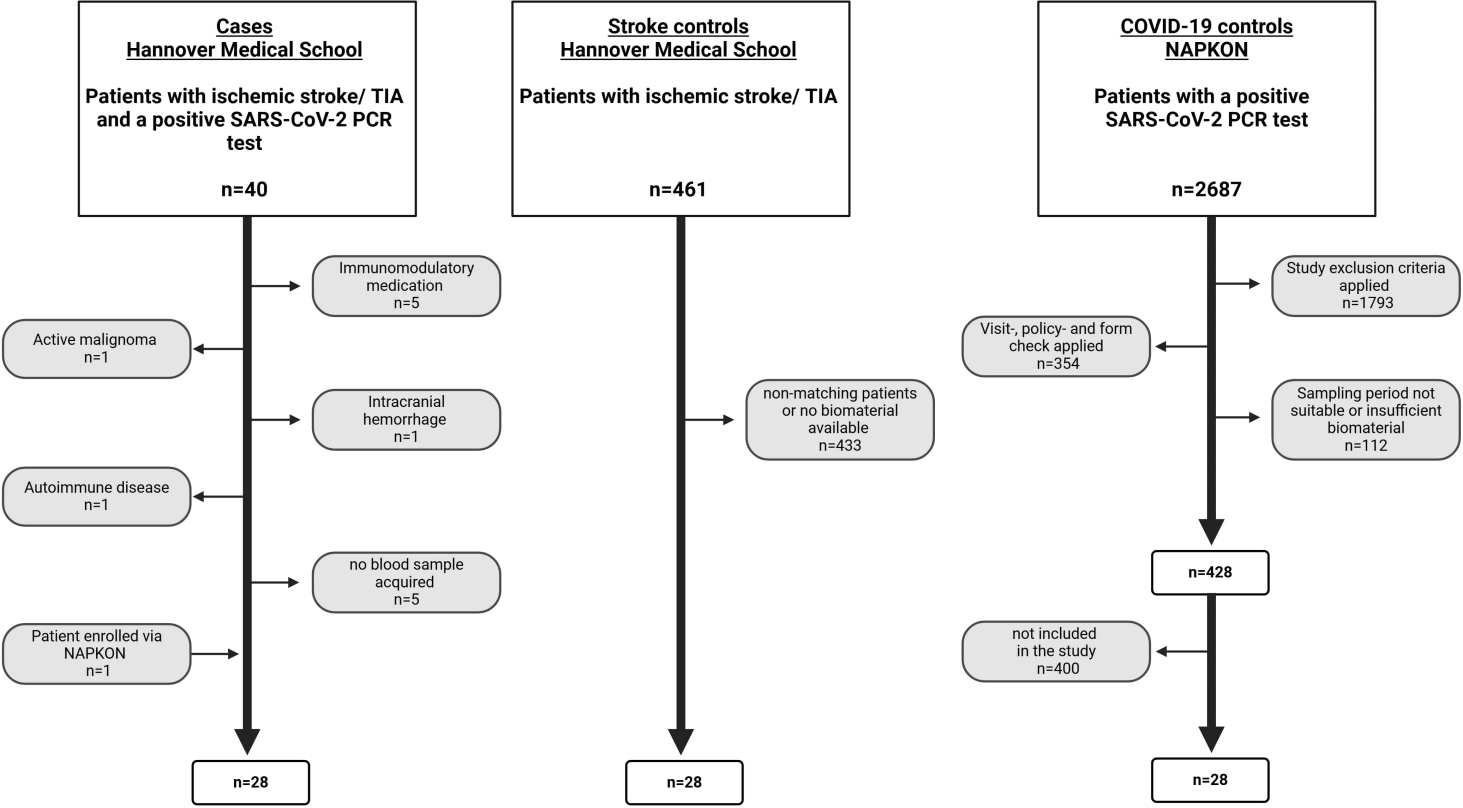
Overview of recruitment process of patients in all groups prior to marker analysis. Created in BioRender. Ernst, J (2025). https://BioRender.com/h99h445.

The first group (n=28, further referred to as *cases*) included patients with acute ischemic stroke or TIA who simultaneously tested positive for SARS-CoV-2. These patients were recruited prospectively at Hannover Medical School between December 2021 and May 2023. Each patient admitted to the stroke unit at Hannover Medical School with a clinically diagnosed ischemic stroke or TIA was screened for SARS-CoV-2 infection using nasopharyngeal swab antigen tests confirmed by PCR within 48 hours of hospitalization in line with a hospital-wide policy mandating universal SARS-CoV-2 testing of all inpatients on admission, irrespective of symptoms. Exclusion criteria across all groups included immunomodulatory therapy, autoimmune diseases, active malignancy, organ transplantation, plasmapheresis, or other conditions affecting immune function (e.g., HIV). For the stroke control group specifically, the presence of acute and chronic infections was an additional exclusion criterion. **Figure 1** provides a detailed overview of the recruitment process.

Of the initially identified 40 patients, 13 were excluded: 7 due to meeting the general exclusion criteria, 5 due to unavailable blood samples, and 1 because a suspected ischemic stroke was later confirmed to be hemorrhagic. One patient from the University Medical Center Göttingen (UMG) met the inclusion criteria and was subsequently included in the study through the National Pandemic Cohort Network (NAPKON).

The second group (n=28, further referred to as *stroke controls*) includes patients with clinically and radiologically confirmed acute ischemic stroke but without concurrent SARS-CoV-2 infection. These patients were recruited during a previous study by our group conducted at Hannover Medical School between August 2014 and November 2018 (14).

A total of 461 patients were screened for eligibility using a comprehensive dataset that included detailed clinical and demographic information, such as age, sex, comorbidities, medication use, stroke related data, treatment details, and the availability of biomaterial. From this cohort, 28 patients were selected to closely match the case group in terms of clinical and demographical characteristics prior to marker analysis.

The third group (further referred to as *COVID-19 controls*) included patients with SARS-CoV-2 infection confirmed by PCR at the time of blood sampling by peripheral venous puncture. Recruitment occurred between January 2021 and March 2023 via the NAPKON network, encompassing multiple clinics across Germany. An initial preselection excluded all patients who did not meet inclusion criteria, met exclusion criteria, or lacked sufficient blood for analysis. From the remaining 428 patients, 28 were selected to closely match the case group in terms of predefined clinical and demographic parameters.

### Demographic and clinical data

The demographical data were collected using standardized Case Report Forms (CRF). For all patients, we collected demographic information and basic clinical parameters. We collected data about pre-existing conditions that are known to increase the risk for stroke or TIA, including obesity, atrial fibrillation, diabetes mellitus, arterial hypertension, history of stroke or TIA, history of myocardial infarction, hyperlipoproteinemia, peripheral arterial disease, coronary artery disease, heart valve defects, chronic heart failure, renal insufficiency, alcohol abuse, inhalative tobacco use, chronic obstructive pulmonary disease (COPD), and asthma. Based on these data, we calculated the Essen Stroke Risk Score (ESRS) for each patient. Additionally, information on medication usage at the time of hospital admission and in the period shortly before was collected for all patients.

For each case and COVID-19 control, we also determined the exact day of symptom onset as well as the severity of COVID-19 disease, using the WHO clinical progression scale (WHO Scale) (15). For each case and stroke control we determined stroke severity at baseline using the National Institutes of Health Stroke Scale (NIHSS) and defined the premorbid modified Rankin scale (mRS) (16). We determined the time between the onset of focal neurological deficits and blood sampling. If the time of onset was unclear, the time at which the patient was last seen well was considered. Moreover, we determined the stroke etiology according to the Trial of Org 10172 in Acute Stroke Treatment (TOAST) (17).

### Matching

Each patient was matched in a 1:1:1 fashion to controls from the stroke control and COVID-19 control groups. Matching in each group was based on age, sex, and pre-existing conditions influencing stroke risk. For matching between cases and stroke controls, stroke severity was also matched using the NIHSS and use of recombinant plasminogen-activator (rt-PA) before blood sampling. Furthermore, the time from neurological symptom onset to blood sampling was considered as a matching criterion between cases and stroke controls. Cases and COVID-19 controls were matched considering the time between symptom onset and blood sample collection, as well as the severity of COVID-19 using the WHO Scale.

### Sample acquisition and measurement of NET marker, regulators and cytokines

In the case group and the stroke control group, blood was drawn via peripheral venous puncture as early as possible after onset of neurological deficits. In the COVID-19 group, blood was collected by peripheral venous puncture at specific time points during the course of the illness following a defined protocol. Blood samples were collected to obtain serum, immediately centrifuged following the manufactures instruction protocol for the serum collection, and the resulting cell-free supernatant was transferred into cryotubes. The samples were subsequently frozen and stored at –80°C until further analysis.

We analyzed all serum samples with commercially available enzyme activity assays or ELISA following the manufacturer’s instructions. The samples were analyzed in two separate runs. In each run half of the samples from all three groups were included. Samples without a measurable value were set to the detection limit of the respective ELISA. Regarding NET-associated markers and regulators, we quantified citrullinated histone H3 (H3cit), neutrophil elastase, myeloperoxidase (MPO) activity, LL-37 and DNase I activity. In order to quantify a broad panel of cytokines, chemokines and growth factors, we used the HCYTA-60K-PX38 Millipore MILLIPLEX^®^ Human Cytokine/Chemokine/Growth Factor Panel. Detailed information on all NET-related assays and the complete list of analytes included in the multiplex panel, including assay kits, sample volumes and experimental conditions, is provided in **Supplementary Tables 1**, **2**.

### Data analysis and statistics

Continuous clinical data, as well as cytokine and NET marker levels, were compared between the case group and each control group using the Mann-Whitney U test.

Binary logistic regression analyses were performed to examine associations between NET marker levels and group-specific conditions. Only a single covariate merging cardiovascular risk factors was included, ensuring an optimal ratio between the number of cases and independent variables. The assumption of linearity of logits was checked, with no violation observed. As only one covariate was included, multicollinearity was not applicable. For the comparison between the case and COVID-19 control group, the presence of an acute ischemic stroke or TIA was used as the dependent variable, with NET marker levels as the independent variable and the ESRS score as a covariate. When comparing the case and stroke control group, SARS-CoV-2 infection served as the dependent variable, again using NET marker levels as the independent variable and adjusting for ESRS by using it as a covariate. Similarly, in the comparison between the stroke control and COVID-19 control group, the presence of a stroke was the dependent variable, with NET marker levels as the independent variable and ESRS as a covariate.

Furthermore, the Spearman rank test was used to explore potential correlations between NET markers and cytokines. Correlation matrices were created for all patients as well as for each subgroup, containing the correlation coefficient rho. The correlation matrices of the subgroups were compared, and the differences between the correlation coefficients were calculated. To improve visual clarity, all results from the correlation matrices were visualized using heatmaps.

If values could not be measured by the applied testing methods due to low concentrations, the missing data was replaced using extrapolation. If more than 50% of the values for a specific marker were unmeasurable across all groups, the marker was excluded from further analyses.

The statistical analysis was performed using IBM SPSS Statistics Version 29.0.2.0 and GraphPad Prism version 10.4.1. The corresponding author had full access to all data in the study and takes responsibility for its integrity and accuracy.

### Ethics

The study was approved by the ethics committee at Hannover Medical School (MHH) (vote no. 9876). Written informed consent to participate in this study was obtained from all patients or, if they were unable to provide consent themselves, from their proxies. All procedures related to this study were conducted in accordance with the Declaration of Helsinki and its later amendments.

## Results

### Clinical and demographic characteristics

**Table 1** presents the clinical and demographic data of all patients. Overall, the three groups (cases, stroke controls, and COVID-19 controls) displayed few differences in their baseline characteristics. The median age of the case group was 69 years while stroke controls had a median age of 72.5 years and COVID-19 controls had a median age of 74 years. One patient in the case group suffered a TIA with a transient sensorimotor hemisyndrome. The vascular risk profile, measured using the ESRS, was comparable between groups, with a median score of 2 for the case group and a median score of 3 for both control groups. Overall, the median time between the onset of neurological symptoms and blood sampling was 17 hours across the case and stroke control groups.

**Table 1:** Clinical and demographic characteristics.

Clinical characteristics	Measure	All patients (n=84 or n=56 if group is N/A)	Patients with stroke or TIA and SARS-CoV-2 (n=28)	Stroke controls (n=28)	COVID-19 controls (n=28)
Age, y	Median (25th-75th percentile)	71 (58.75–80.25)	68.5 (57–77.25)	72.5 (62.25–81)	74 (60.5–81)
ESRS	Median (25th-75th percentile)	3 (1.75–4)	2 (1–3)	3 (2–4)	3 (1–4)
NIHSS Day 1	Median (25th-75th percentile)	4 (2–13)	3 (1.5–13.5)	4.5 (2–13)	N/A
NIHSS Day 7 or on day of discharge	Median (25th-75th percentile)	2 (0–4)	1 (0–3.75)	2 (1–4)	N/A
mRS 0d (before baseline)	Median (25th-75th percentile)	0 (0–1)	0 (0–0)	0 (0–1)	N/A
mRS 90d (after baseline)	Median (25th-75th percentile)	2 (1–3)	1 (0.5–3)	2 (1–3)	N/A
Sex male	n%	60 (71.4)	20 (71.4)	20 (71.4)	20 (71.4)
Sex female	n%	24 (28.6)	8 (28.6)	8 (28.6)	8 (28.6)
BMI	Median (25th-75th percentile)	27.25 (24.83–30.68)	25.65 (24.58–27.73)	29.4 (24.65–30.95)	28.15 (25.93–31.08)
Patients showing or showed COVID-19 related symptoms	n%	43 (76.79)	15 (53.57)	N/A	28 (100)
COVID-19 symptom onset time detectable	n%	42 (75)	14 (50)	N/A	28 (100)
Interval between COVID-19 symptom onset and blood sampling, d	Median (25th-75th percentile)	8.5 (6.25–14)	7 (2.25–15)	N/A	9.5 (7–13.25)
Unknown time of stroke onset	n%	11 (19.64)	4 (14.29)	7 (25)	N/A
Severity of COVID-19 disease according to WHO scale	Median (25th-75th percentile)	4 (4–5)	4 (4–4.25)	N/A	4.5 (4–5)
Intravenous rt-PA thrombolysis prior blood-sampling	n%	19 (33.93)	9 (32.14)	10 (35.71)	N/A
Atrial fibrillation	n%	21 (25)	8 (28.57)	7 (25)	6 (21.43)
Obesity (BMI > 30)	n%	24 (28.57)	4 (14.29)	11 (39.29)	9 (32.14)
Diabetes	n%	18 (21.43)	5 (17.86)	6 (21.43)	7 (25)
Arterial hypertension	n%	57 (67.86)	18 (64.29)	23 (82.14)	16 (57.14)
Previous stroke or TIA	n%	8 (9.52)	2 (7.14)	5 (17.86)	1 (3.57)
Previous myocardial infarction	n%	5 (5.95)	1 (3.57)	1 (3.57)	3 (10.71)
Hyperlipoproteinemia	n%	27 (32.14)	14 (50)	8 (28.57)	5 (17.86)
Peripheral arterial occlusive disease	n%	9 (10.71)	3 (10.71)	4 (14.29)	2 (7.14)
Coronary heart disease	n%	14 (16.67)	3 (10.71)	2 (7.14)	9 (32.14)
Heart valve defects	n%	11 (13.10)	5 (17.86)	2 (7.14)	4 (14.29)
Chronic heart failure	n%	8 (9.52)	4 (14.29)	1 (3.57)	3 (10.71)
Chronic kidney disease	n%	16 (19.05)	4 (14.29)	7 (25)	5 (17.86)
Alcohol addiction	n%	6 (7.14)	1 (3.57)	3 (10.71)	2 (7.14)
Lung disease (COPD, Asthma)	n%	10 (11.90)	2 (7.14)	2 (7.14)	6 (21.43)
Smoker	n%	31 (36.90)	9 (32.14)	11 (39.29)	11 (39.29)
Stroke etiology (according to TOAST)					
Large artery atherosclerosis	n%	7 (12.50)	3 (10.71)	4 (14.29)	N/A
Cardioembolic stroke	n%	20 (35.17)	9 (32.14)	11 (39.29)	N/A
Small vessel disease	n%	3 (5.36)	2 (7.14)	1 (3.57)	N/A
Dissection, vasculitis, other etiology	n%	3 (5.36)	2 (7.14)	1 (3.57)	N/A
Cryptogenic stroke	n%	15 (26.79)	4 (14.29)	11 (39.29)	N/A
Diagnostic workup incomplete	n%	8 (14.29)	8 (28.57)	0 (0)	N/A

BMI, Body mass index, COPD, Chronic obstructive pulmonary disease, ESRS, Essen stroke risk score, mRS, modified Rankin scale, NIHSS, National Institute of Health Stroke Scale, rt-PA, recombinant tissue plasminogen activator.

It was observed that patients in the COVID-19 control group more frequently exhibited COVID-19-typical symptoms such as fever, cough, rhinitis, and myalgia. In contrast, patients in the case group were more often asymptomatic (n=15 vs. n=28) and frequently did not present with typical COVID-19 symptoms. This led to the issue that determining an exact onset of infection or symptoms was not always possible. As a result, the calculations of the time intervals were based solely on the data from patients with an identifiable onset of COVID-19 symptoms. Regarding the COVID-19 severity measured by WHO scale, it should be noted that no relevant clinical differences were found between the case group and the COVID-19 control group. However, it is important to mention that patients were strictly classified according to the WHO scale, meaning that a hospital admission combined with a positive PCR test automatically resulted in a WHO scale of 4, even if the admission was not primarily due to COVID-19 related symptoms.

In some patients in the case group no differential diagnostic workup could be performed because of the necessity of isolation measures due to SARS-CoV-2 infection and the associated precautionary measures within the hospital. Following neurological improvement, some patients with a persistently positive SARS-CoV-2 test were discharged to home quarantine. As a result, in 8 patients in the case group a differential diagnostic workup was not completely performed and the etiology could not be clearly identified.

### NET analyses

**Figure 2:**
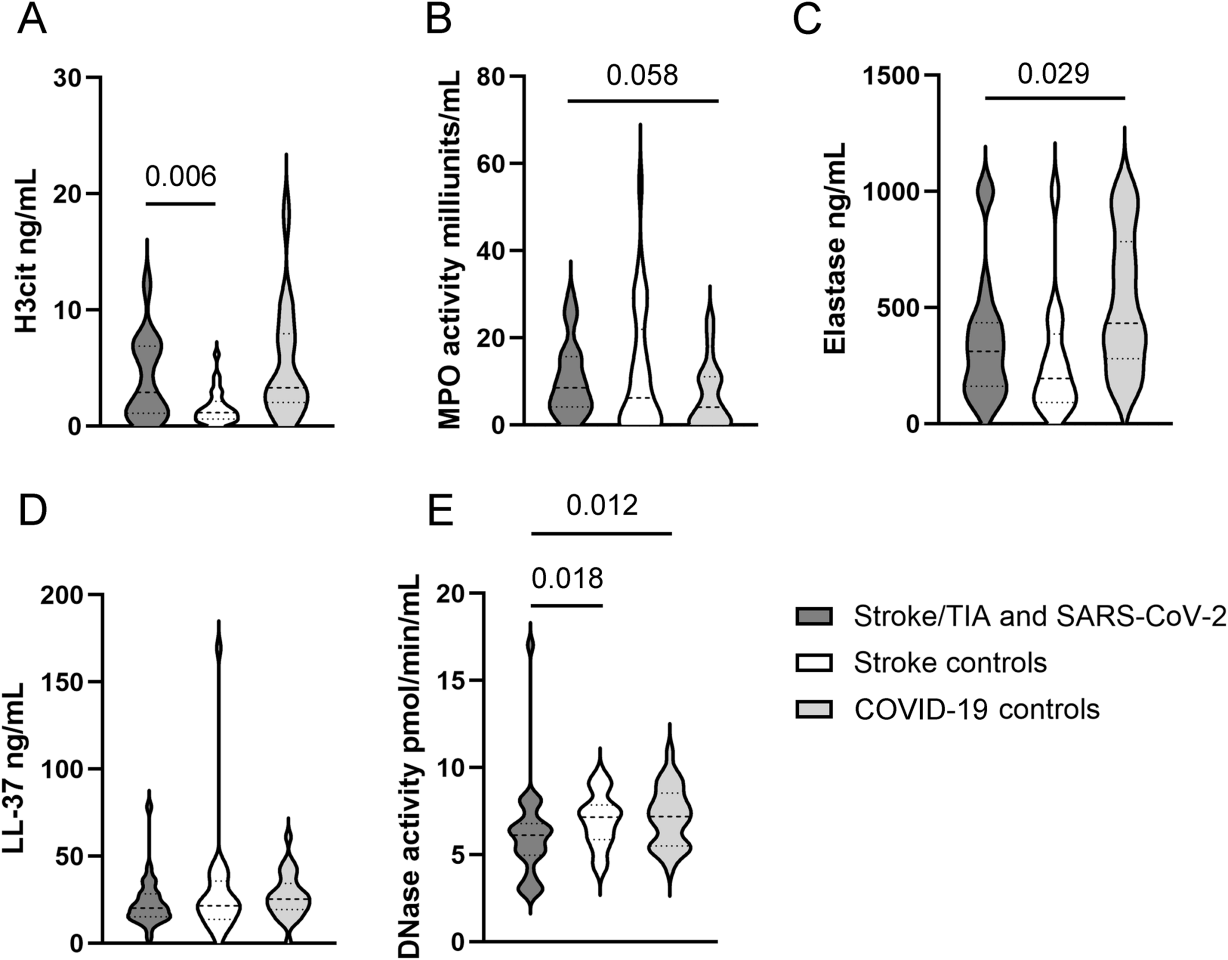
**(A-E)** Violin plots showing the distribution of NET associated markers across the three study groups. The quartiles are represented by horizontal dotted lines, the median is indicated by a horizontal dashed line within the plot. Data were analyzed with two-tailed Mann-Whitney test calculated always to Stroke/TIA and SARS-CoV-2 group. This figure was created using GraphPad Prism Version 10.4.1.

The results of the NET marker and regulator measurements across all three groups are presented in **Figures 2A–E** using violin plots. The given p values in this paragraph are derived from the Mann-Whitney-U test. Median and quartiles of NET marker measurements can be found in **Supplementary Table 3** in the **Supplementary Material**. The concentration of H3cit was considerably higher in the case group compared to the stroke control group (p=0.006). Likewise, it was substantially higher in the COVID-19 control group than in the stroke control group (p<0.001). Elastase was highest in the COVID-19 control group, showing a clear difference compared to the case group (p=0.029) and the stroke control group (p<0.001). Modest differences were found between the groups in terms of MPO activity. The MPO activity in the case group tended to be higher than in the COVID-19 control group (cases vs. COVID-19 controls: p=0.058) with no difference observed between the case group and the stroke control group (cases vs. stroke controls: p=0.465) and between stroke controls and COVID-19 controls (p=0.417). However, it should be noted, that in 18 of 84 patients the MPO activity was below the detection limit, this might have an impact on the results. Similarly, no relevant differences were observed in the concentration of LL-37 (cases vs. stroke controls: p=0.640, cases vs. COVID-19 controls: p=0.123, stroke controls vs. COVID-19 controls: p=0.385). A notable difference was observed in DNase activity, which was lower in the case group compared to the stroke control group (p=0.018) and also lower than in the COVID-19 control group (p=0.013). DNase activity did not differ between the stroke control and COVID-19 control groups (p=0.768).

We conducted a binary logistic regression analysis to adjust for confounding despite matching. For the comparison between the case and the COVID-19 control group, the dependent variable was defined as the presence of stroke or TIA, with the respective measured NET markers as independent variables. Age and cardiovascular comorbidities, combined in the ESRS score, were included as covariates. For comparison between the case and the stroke control group, the presence of SARS-CoV-2 infection was defined as the dependent variate and the measured NET markers served as independent variables. For adjustment, the ESRS was used as a covariate. For all markers, specific values for crude and ESRS-adjusted odd ratios can be found in **Table 2**. After adjustment for ESRS, the differences in DNase activity between the groups were less pronounced compared to the initial analysis using the Mann-Whitney U test. The direct comparison between the case group and the stroke control group revealed no relevant differences of the crude (OR = 1.242; 95% CI 0.93–1.658; p=0.143) and the adjusted (OR = 1.237; 95% CI 0.924–1.657; p=0.153) values. A similar pattern was observed when comparing the case group to the COVID-19 control group (Crude OR = 1.279; 95% CI 0.961–1.704; p=0.092; Adjusted OR = 1.323; 95% CI 0.979–1.788; p=0.069). In addition, only minor differences in H3cit levels, elastase levels, MPO activity and LL-37 levels were present across all groups after adjustment for ESRS.

**Table 2:** Crude and ESRS-adjusted Odds-ratios.

Marker	Cases vs. stroke controls				Cases vs. COVID-19 controls			
	Crude OR (95% CI)	P-value	Adjusted (ESRS) OR (95% CI)	P-value	Crude OR (95% CI)	P-value	Adjusted (ESRS) OR (95% CI)	P-value
DNase activity	OR 1.242 (0.93–1.658)	0.143	OR 1.237 (0.924–1.657)	0.153	OR 1.279 (0.961–1.704)	0.092	OR 1.323 (0.979–1.788)	0.069
MPO activity	OR 1.01 (0.965–1.057)	0.660	OR 1.008 (0.96–1.058)	0.762	OR 0.941 (0.876–1.011)	0.097	OR 0.945 (0.879–1.017)	0.131
H3cit	OR 0.664 (0.5–0.881)	0.005	OR 0.69 (0.516–0.923)	0.012	OR 1.082 (0.949–1.234)	0.239	OR 1.11 (0.966–1.276)	0.141
Elastase	OR 0.999 (0.996–1.001)	0.198	OR 0.999 (0.997–1.001)	0.306	OR 1.002 (1.000–1.004)	0.039	OR 1.002 (1.000–1.004)	0.038
LL-37	OR 1.01 (0.984–1.038)	0.445	OR 1.014 (0.988–1.042)	0.289	OR 1.021 (0.979–1.064)	0.337	OR 1.028 (0.984–1.074)	0.213

(OR) for NET marker comparison between the groups with corresponding 95% confidence intervals and p-values.

### Cytokine analyses

Several cytokines were excluded from further analyses due to over 50% being non-measurable across all groups. These cytokines include GM-CSF, IFNα2, IFNγ, IL-1α, IL-1β, IL-2, IL-3, IL-12 (p70), IL-13, IL-17A, IL-17E/IL-25, IL-22, MIP-1α, and TNF-β.

**Figure 3:**
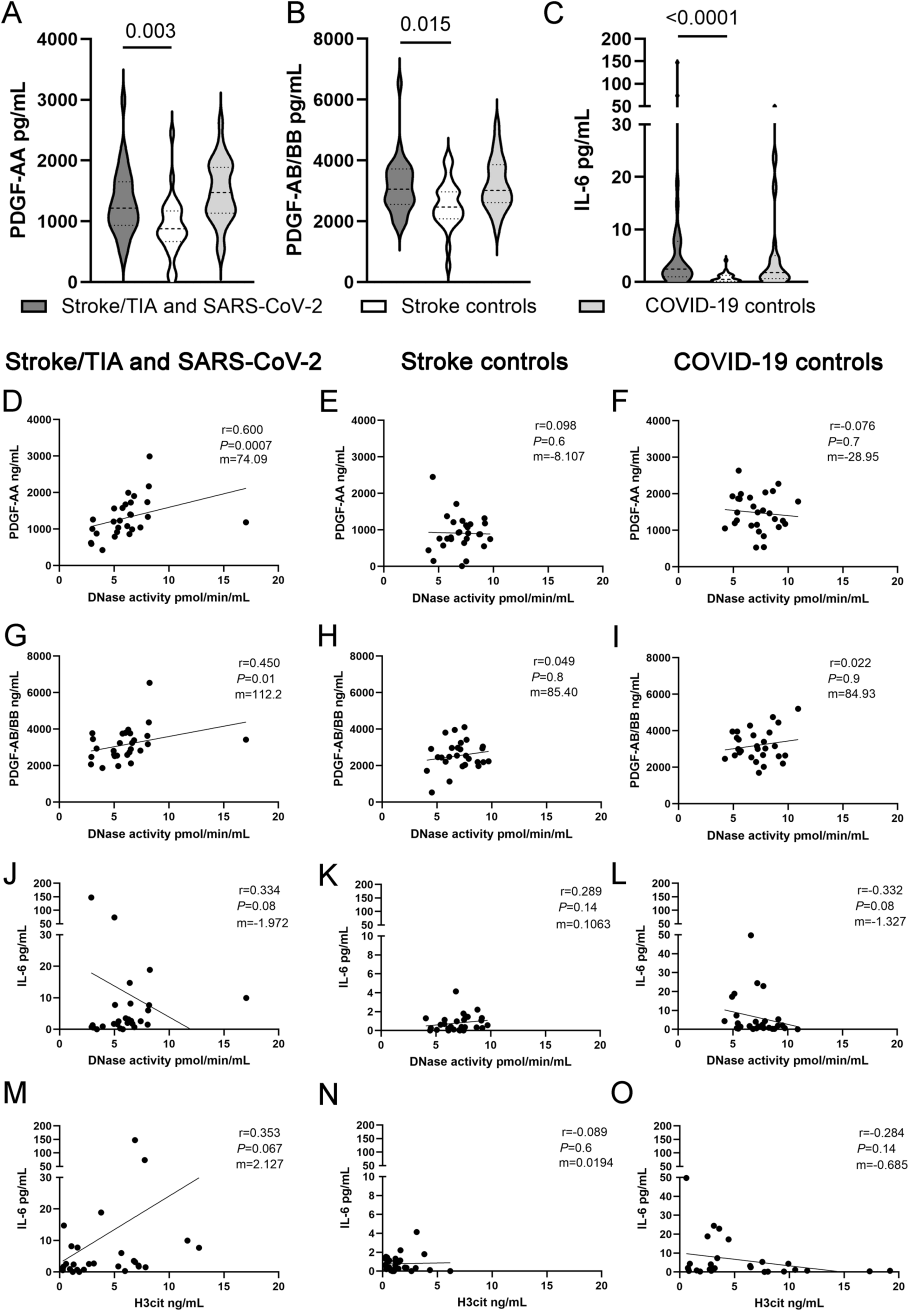
Violin plots illustrating groupwise differences and correlations between cytokines and NET markers. **(A–C)** Violin plots showing the distribution of cytokine levels (PDGF-AA, PDGF-AB/BB and IL-6) across the three study groups. The quartiles are represented by horizontal dotted lines, the median is indicated by a horizontal dashed line within the plot. Data were analyzed with two-tailed Mann-Whitney test calculated always to Stroke/TIA and SARS-CoV-2 group. **(D–O)** Scatter plots depicting correlations between selected cytokines and NET-associated markers, analyzed separately for each group. Correlations were assessed using Spearman’s rank correlation coefficient. This figure was created using GraphPad Prism Version 10.4.1.

Median and quartiles of cytokine measurements can be found in **Supplementary Table 4** in the **Supplementary Material**. The cytokine profiles of the case group and the COVID-19 control group were largely comparable. In contrast, notable disparities were evident when either group was compared to the stroke control group. Specifically, we observed that PDGF-AA and PDGF-AB/BB, as well as proinflammatory cytokines (e.g. IL-6) were significantly higher in SARS-CoV-2 infected patients of the case group compared to the stroke control group (see **Figures 3A–C**).

To explore potential correlations between cytokines and NET markers, correlation coefficients were calculated using the Spearman-rank test. Initially, we analyzed intra-group correlations, followed by correlations across all groups. Also, we calculated the difference between two ρ-coefficients between all subgroups and visualized these differences graphically. The correlation matrices and the results of this comparison are shown in detail in **Supplementary Figures 1a–f**. The results are presented using a color-coded heatmap. The most pronounced differences were observed between the case and COVID-19 control groups regarding the correlation of cytokines with DNase activity. In the case group various cytokines tended to correlate significant positively with DNase activity, but not in the control groups (**Figures 3D–L**). Particularly strong correlations were identified for PDGF-AA and PDGF-AB/BB: ρ=0.6 and 0.45, p=0.001 and 0.016, respectively (**Figures 3D, G**). A tendency of positive correlation was observed for IL-6 in the case group (**Figure 3J**). In contrast, in the COVID-19 control group, IL-6 showed a negative correlation with DNase activity (**Figure 3L**), A similar pattern was seen for H3cit and IL-6, with significant positive correlation in the case group, which was not observed in the control groups (**Figures 3M–O**). Correlations across all NET markers and cytokines can be found in the **Supplementary Material** in **Supplementary Figures 2a–d**.

## Discussion

This pilot study investigated the immunological mechanisms underlying SARS-CoV-2-associated cerebral ischemia, focusing on the role of NETs and cytokines. Our results suggest that patients with acute COVID-19 and ischemic stroke exhibit distinct patterns of NET formation, degradation and cytokine profiles compared to non-stroke COVID-19 controls and non COVID-19 stroke patients, indicating a potential pathogenic link between dysregulated innate immunity and cerebrovascular complications.

The observed elevation of NETs and specific cytokines in the case group aligns with recent evidence suggesting that immunothrombosis is a key mediator of stroke in viral infections, particularly SARS-CoV-2. As recently a systematic review and meta‐analysis of 155 studies identified that different viral infections increase the risk of major cardiovascular events, including stroke (18), our results shade light on the pathogenesis of “viral induced stroke”. Several studies have indicated the involvement of NETs in ischemic stroke thrombi and their direct relation to clinical outcome (19, 20). Additionally, it has been shown that SARS-CoV-2 leads to elevated NET markers and specific cytokines in the blood of patients (9, 11). We observed higher values for H3cit and elastase in patients with SARS-CoV-2 infection compared to those without (**Figure 2**), indicating a higher amount of NETs presumably as an immunological response to viral infection. A notable result in our cohort is the reduction of DNase activity in patients with acute ischemic stroke or TIA during SARS-CoV-2 infection (**Figure 2e**). DNase can degrade cell-free DNA released during NETosis, thereby possibly reducing the formation of scaffolds that support the aggregation of prothrombotic substances (21). Impaired DNase-mediated degradation of NETs has been demonstrated in patients with thrombotic microangiopathies, where persistent NETs due to reduced plasma DNase activity were suggested to contribute to microvascular thrombosis (22). Given similar levels of NET markers in SARS-CoV-2-positive patients, it may be hypothesized that the reduced DNase activity in the group with concomitant stroke could lead to an accumulation of NETs, potentially contributing to immunothrombosis. Therefore, it can be speculated that impaired NET degradation is a reason for cerebral ischemia during SARS-CoV-2 infection. In the present study there may be variability in the duration and timing of infection onset relative to cerebrovascular events in the subset of asymptomatic individuals. This residual heterogeneity may influence immunological markers such as NETs and cytokines and must be considered when interpreting the results. However, when considering patients with known and without known symptomatology from SARS-CoV 2 infection we did not observe differences in DNase activity. It has been suggested that the external application of DNase could serve as a potential therapeutic approach for ischemic stroke treatment (23–25). In a current phase 2 study, the clinical use of recombinant DNase-1 as an adjunct to established therapy with rt-PA in intravascular thrombolysis is being investigated and another one is planned to test DNase for secondary stroke prevention (26, 27). Our data also suggest that higher levels of DNase activity may offer protection against ischemic stroke or TIA during acute SARS-CoV-2 infection. The current studies on the therapeutic application of DNase in thrombolytic regimens further underscore the clinical relevance of this pathway. However, it remains unclear whether individuals with reduced DNase activity inherently produce insufficient amounts of the enzyme, or whether its depletion occurs as a consequence of an antiviral response or an ongoing ischemic event like ischemic stroke. There is evidence suggesting various components may impact endogenous DNase activity, including genetic factors (28), the presence of acute and chronic inflammation (29) as well as sex (30).

**Figure 4:**
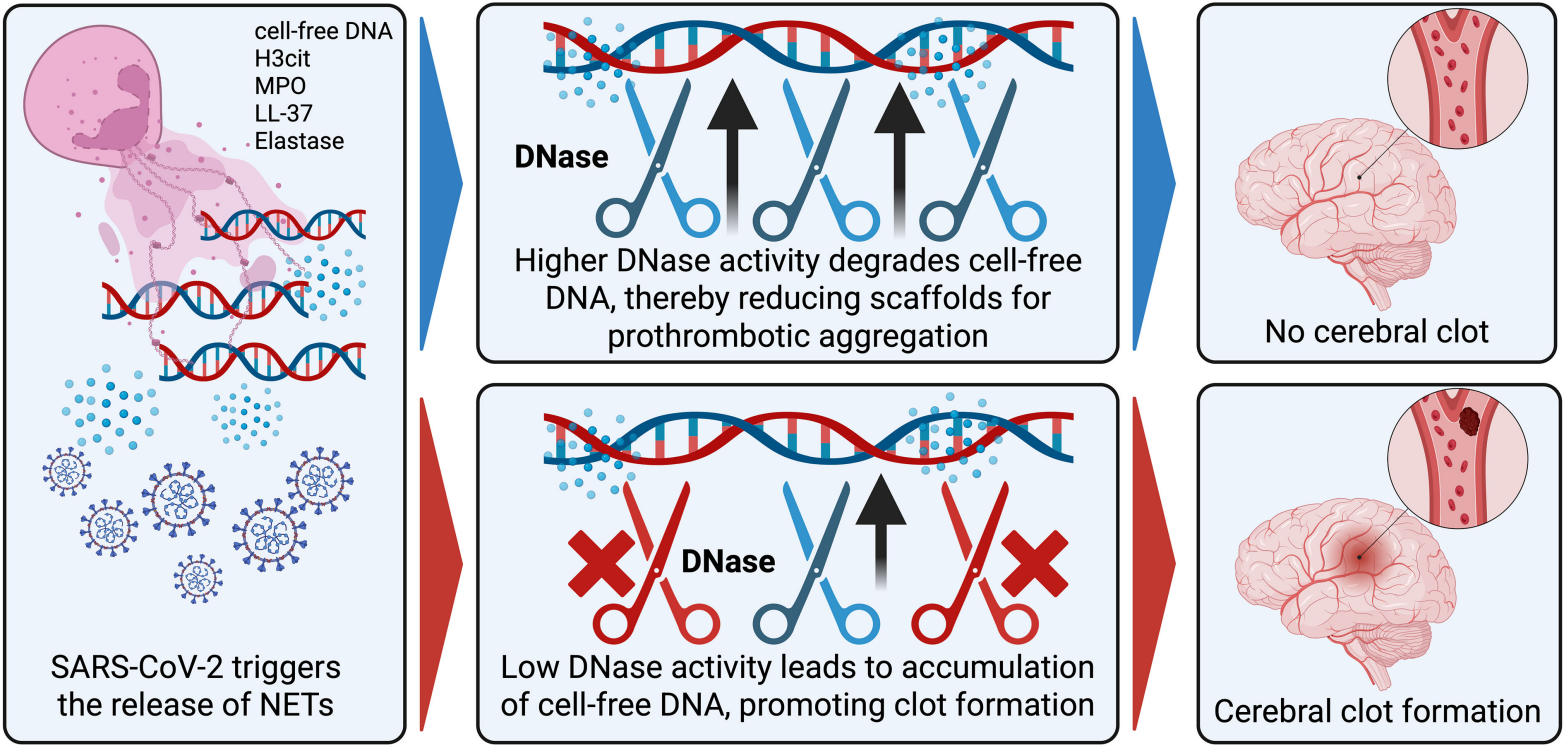
Proposed pathophysiological model linking NET formation, impaired DNase activity, and SARS-CoV-2–associated stroke. Created in BioRender. Plem, R. (2025) https://BioRender.com/7un9lkn.

Our data highlight that the mechanism of immunothrombosis—potentially fueled by increased NET production and inadequate clearance—may be relevant in stroke associated with SARS-CoV-2. However, other viral illnesses, such as influenza and herpesviruses, have also been linked to short-term stroke risk, even in pediatric populations (31–34). Given that infections with viruses like influenza lead to higher amounts of NET associated biomarkers in the blood (8), it may be hypothesized that NETosis, combined with insufficient resolution of these structures—such as through reduced DNase activity —could explain why certain patients are at greater risk of immunothrombosis following viral infections. **Figure 4** provides a schematic overview of the proposed pathophysiological mechanisms involving NET formation and DNase activity in SARS-CoV-2–associated stroke. The interplay between proinflammatory cytokines and NET dynamics appears to further shape the immunothrombotic landscape in COVID-19-related stroke. Recent studies indicate that various cytokines—including TNF-α, IL-1β, and IL-8—not only modulate neutrophil activation, but also directly enhance NET formation in sterile and infectious inflammatory states (35). This complex relationship suggests that cytokine signaling could alter both NET production and clearance. Building on this concept, we explored the correlations between cytokine profiles and NET marker/DNase activity in our cohort.

In the case group, strong positive correlations were found between DNase activity and IL-6, and PDGF-AA/PDGF AB-BB (**Figures 3D, G, J**). In contrast, the COVID-19 control group showed negative correlations (**Figures 3F, I, L**). Taking into consideration that previous studies (21) showed an inverse correlation between DNase and proinflammatory cytokines (TNF-alpha, IL-6, IFN-γ) on day 7 after stroke, we observed the same correlation within the COVID-19 control group at baseline, but an opposite pattern in the case group. This suggests that COVID-19-associated strokes may represent a unique immunothrombotic phenotype, in which the regulation of NET degradation and cytokine signaling are distinctly altered. The most pronounced correlation of cytokines and NETs markers in the case group could be detected for DNase activity and PDGF-AA/PDGF AB-BB (**Figures 3D, G**). In a previous work from our group, we identified PDGF-AB/BB as independent predictor of insufficient reperfusion status (36). PDGF also exerts chemotactic effects on neutrophils and monocytes and—alongside various other cytokines and chemokines—likely contributes to leukocyte recruitment at the site of the thrombus, making it a compelling target in thromboinflammatory processes (37, 38). In an analysis of multiple inflammatory cytokines and growth factors, Kim et al. identified PDGF-AB/BB as the sole predictor of intracranial stenosis progression, underscoring its importance in atherosclerotic pathology (39). On the other hand, high levels of PDGF AA have been described in COVID-19 patients (40). Beyond these findings, PDGF may play a particularly relevant role in the interplay between NET regulation, infection-related inflammatory activation, and cerebrovascular events, suggesting its broader involvement in immune–vascular crosstalk during acute and chronic vascular injury. Future studies could characterize this phenotype in more detail.

Our findings also fit into a broader framework in which insufficient NET clearance promotes the formation of anti-NET autoantibodies, endothelial injury and self-amplifying inflammatory–thrombotic circuits. In autoimmune diseases such as systemic lupus erythematosus, NETs can contribute to pathogenesis through self-amplifying vicious cycles (41). A key element in this process is the presence of autoantibodies directed against components of NETs, such as MPO, PR3, double-stranded DNA or histones (41, 42). These autoantibodies not only trigger NET-formation via immune complexes but also impair NET degradation by interfering with DNase activity or by binding and stabilizing NETs, preventing their enzymatic breakdown (41–44). This results in the accumulation of NETs, which can further stimulate the immune system, perpetuating chronic inflammation (43). Furthermore, endothelial cells are relevant in this context, as they are vulnerable to NET-induced cytotoxicity and are able to promote further NET-formation when activated (44). This bidirectional interaction may establish a self-amplifying loop, whereby persistent NETs perpetuate endothelial activation and dysfunction (44). A reduction in DNase activity, as observed in our study, can lead to the persistence of cell-free DNA, which may in turn promote neutrophil and endothelial activation and, over time, the development of anti-NET autoantibodies (43). This may initiate a feedback loop in which impaired clearance, endothelial dysfunction and increased NET-formation mutually reinforce each other.

This study has strengths and limitations. A particular strength of this work is the good comparability between the respective subgroups due to the similar demographic and clinical characteristics of the patients. Moreover, the comprehensive overview of clinical characteristics and biomarkers enables us to detect subtle distinctions between the groups, informing a more precise interpretation of our findings. The endogenous response towards the infection with SARS-CoV-2 was the main focus of our work. Therefore, matching with regard to the severity of infectious symptoms enabled us to investigate not merely the association between SARS-CoV-2 and stroke, but also the effect of the differential endogenous response to infection. There are also some limitations to this work. [1] The small sample size represents an inherent limitation of our study. However, this pilot approach enabled us to establish feasibility, demonstrate methodological robustness, and generate preliminary insights into the immunological landscape of SARS-CoV-2-associated stroke. These findings provide an important basis for future, adequately powered studies. [2] Also, there is potential for inaccuracies due to the fact that the interval between the onset of COVID-19 symptoms and blood sampling could not be determined for asymptomatic patients with a positive SARS-CoV-2 PCR test. It was not possible for us to recruit asymptomatic SARS-CoV-2-positive patients as controls, as they rarely sought medical attention due to a lack of symptoms. As a result, in the COVID-19 control group, we included only patients who exhibited COVID-19 specific symptoms, whereas in our case group, several patients exhibited barely any infection-like symptoms at all. [3] The recruitment periods between groups were not fully matched, which could introduce temporal confounding due to changes in circulating SARS-CoV-2 variants, evolving treatment protocols, or population immunity. While both periods largely overlapped with Omicron predominance, we cannot entirely rule out a bias from temporal factors. [4] In 18 out of 84 patients, MPO activity was below the detection limit, which likely contributed to the unexpected lack of difference in MPO activity between SARS-CoV-2 positive and negative patients. This may be due to the use of an activity-based assay rather than quantification of total MPO protein levels. This methodological limitation should be considered in future studies. [5] It is well known that the inflammatory activity of COVID-19 varies depending on the stage of the disease. This variation in disease activity is likely to influence NETosis as well. Although we attempted to match patients based on symptom severity and disease stage, this was only partially achievable due to the aforementioned limitations.

In conclusion, biomarkers of NETosis and its regulation are altered in stroke associated with SARS-CoV-2. Specifically, we were able to suggest differences in DNase activity and DNase-cytokine correlations in patients infected with SARS-CoV-2 and acute ischemic stroke and TIA compared to patients with acute ischemic stroke without SARS-CoV-2 infection and to those with SARS-CoV-2 infection but without acute ischemic stroke. Considering that similar mechanisms may be involved in the immune response to other viral infections, further research in studies with larger sample sizes and other infectious diseases is warranted.

## Data Availability

The original contributions presented in the study are included in the article/**Supplementary Material**. Further inquiries can be directed to the corresponding authors.
