# Supplementary material for: Disturbed regulation of immunothrombosis in cerebral ischemia associated with SARS-CoV-2 infection
Source: Front Immunol. 2026 Jan 29;17:1662418. doi: 10.3389/fimmu.2026.1662418 (PMC12894018; doi:10.3389/fimmu.2026.1662418)
Supplement: Supplementary file 1 [file DataSheet1.pdf]

Supplemental Material

STable 1: Overview of NET-associated markers, assay kits and measurement conditions.

Analyte/marker	Assay type	Kit name	Manufacturer (city, country)	Catalogue no.	Sample volume per well*	Sample incubation time*
**DNase I Activity**	Colorimetric activity assay	DNase I Activity Assay Kit	Abcam, Berlin, Germany	ab234056	25 µL	90 min
**Myeloperoxidase (MPO) Activity**	Colorimetric activity assay	Myeloperoxidase Colorimetric Activity Assay Kit	Sigma Aldrich, St. Louis, Missouri, USA	MAK068	10 µL	30 min
**Neutrophil elastase**	ELISA	Human PMN (Neutrophil) Elastase ELISA Kit	Invitrogen, Carlsbad, California, USA	BMS269	10 µl	60min

**Citrullinated histone H3**	ELISA	Citrullinated histone 3 (Clone 11D3) ELISA Kit (H3cit)	Cayman Chemical, Michigan, USA	501620	50µl	60 min
**LL-37**	ELISA	LL-37 ELISA Kit	HycultBiotech, Uden, The Netherlands	HK321-02	10µl	60 min

STable 1 legend: Assays, commercial kits and experimental conditions used for the quantification of NET-associated markers (DNase I activity, MPO activity, neutrophil elastase, H3cit and LL-37). *: assays performed according to the manufacturers´ instructions.

STable 2: Analytes included in the Luminex-based HCYTA-60K-PX38 Millipore MILLIPLEX® Human Cytokine/Chemokine/Growth Factor Panel

**Analyte (full name)**	**Abbreviation / alternative name**
Epidermal growth factor	EGF
Interleukin-1β	IL-1β
Eotaxin	CCL11
Granulocyte colony-stimulating factor	G-CSF
Granulocyte-macrophage-colony-stimulating factor	GM-CSF
Fractalkine	CX3CL1
Interferon-α2	IFN-α2
Interferon-γ	IFN-γ
Interleukin-10	IL-10
Interleukin-12p40	IL-12p40
Interleukin-12p70	IL-12p70
Interleukin-13	IL-13
Interleukin-15	IL-15
Interleukin-17A	IL-17A
Interleukin-1 receptor antagonist	IL-1RA
Interleukin-1α	IL-1α
Interleukin-2	IL-2
Interleukin-3	IL-3
Interleukin-4	IL-4
Interleukin-5	IL-5
Interleukin-6	IL-6

Interleukin-7	IL-7
Interleukin-8	CXCL8
IFN-γ–induced protein 10	IP-10/CXCL10
Monocyte chemotactic protein 1	MCP-1 / CCL2
Macrophage inflammatory protein 1α	MIP-1α / CCL3
Macrophage inflammatory protein 1β	MIP-1β / CCL4
Tumor necrosis factor-α	TNF-α
Tumor necrosis factor-β	TNF-β
Vascular endothelial growth factor A	VEGF-A
Interleukin-17E / Interleukin-25	IL-17E / IL-25
Interleukin-17F	IL-17F
Interleukin-18	IL-18
Interleukin-22	IL-22
Macrophage colony-stimulating factor	M-CSF
Monokine induced by IFN-γ	MIG / CXCL9
Platelet-derived growth factor AA	PDGF-AA
Platelet-derived growth factor AB/BB	PDGF-AB/BB

STable 2 legend: Concentrations of cytokines and growth factors were measured using the Luminex-based HCYTA-60K-PX38 Millipore MILLIPLEX® Human Cytokine/Chemokine/Growth Factor Panel in Serum according to the manufacturer's instructions

STable 3: Overview of the measured levels and activity of NET markers.

Marker	Value	Case group	Stroke control group	COVID-19 control group	p-values		
					Case group vs. Stroke control group	Case group vs. COVID-19 control group	Stroke controls vs. COVID-19 controls
DNase activity (pmol/min/mL)	Median (1.-3. Quartile)	6.124 (4.97–6.784)	7.16 (5.88–7.853)	7.192 (5.515–8.541)	p=0.018	p=0.013	p=0.768
MPO activity (milliunits/mL)	Median (1.-3. Quartile)	8.587 (4.154–15.685)	6.247 (0–21.973)	4.132 (0–11.105)	p=0.465	p=0.058	p=0.417
H3cit (ng/mL)	Median (1.-3. Quartile)	2.902 (1.112–6.889)	1.172 (0.605–2.145)	3.312 (2.031–7.969)	p=0.006	p=0.251	p<0.001
Elastase (ng/mL)	Median (1.-3. Quartile)	312.05 (161.975–435.375)	195.1 (91.99–386.9)	433.1 (280.975–783.75)	p=0.082	p=0.029	p<0.001
LL-37 (ng/mL)	Median (1.-3. Quartile)	20.255 (15.18–28.493)	21.535 (13.818–35.695)	25.385 (19.358–34.405)	p=0.640	p=0.123	p=0.385

STable 3 legend: Median values and interquartile ranges for measured NET-markers for each subgroup with corresponding p-values.

STable 4: Overview of measured cytokine and chemokine levels.

Marker	Cases	Stroke controls	COVID-19 controls
EGF (pg/mL)	4.315 (1.91–19.845)	1.91 (1.91–7.37)	35.4 (23.823–63.64)
Eotaxin (pg/mL)	47.52 (32.965–72.05)	39.055 (26.443–47.783)	44.55 (33.708–72.21)
Fractalkine (pg/mL)	21.015 (5.1–59.6)	10.47 (5.1–18.01)	38.38 (18.01–60.43)
G-CSF (pg/mL)	8.21 (3.38–12.875)	3.05 (0.08–7.003)	9.61 (3.463–21.905)
IL-1RA (pg/mL)	1.14 (0.33–4.83)	0.29 (0.04–0.803)	2.73 (1.44–7.19)
IL-4 (pg/mL)	0.195 (0.105–0.5)	0.135 (0.005–0.205)	0.36 (0.158–0.79)
IL-5 (pg/mL)	0.445 (0.27–0.59)	0.27 (0.123–0.62)	0.615 (0.383–0.87)
IL-6 (pg/mL)	2.45 (0.998–7.733)	0.485 (0.135–1.25)	1.81 (0.63–5.048)
IL-7 (pg/mL)	1.705 (0.865–2.593)	1.27 (0.798–1.858)	1.99 (1.14–3.083)
IL-8 (pg/mL)	6.8 (4.385–8.393)	4 (3.265–6.593)	8 (4.863–13.305)
IL-10 (pg/mL)	3.265 (1.483–5.528)	0.92 (0.585–1.435)	5.895 (2.69–14.705)
IL-12(p40) (pg/mL)	4.995 (2.505–12.638)	0.69 (0.69–2.895)	9.045 (4.62–13.705)
IL-15 (pg/mL)	2.625 (1.76–4.208)	1.7 (1.33–2.178)	3.455 (2.368–5.19)

IL-17F (pg/mL)	2.8 (1.68–4.545)	0.96 (0.61–1.958)	3.765 (2.42–8.675)
IP-10 (pg/mL)	40.285 (22.788–88.503)	18.915 (10.343–23.205)	203.86 (47.748–746.64)
MCP-1 (pg/mL)	159.86 (117.7–256.365)	149.365 (90.71–198.285)	247.47 (150.895–314.645)
M-CSF (pg/mL)	22.17 (11.145–32.73)	7.2 (3.51–13.75)	40.31 (21.71–52.3)
MIG (pg/mL)	219.045 (110.053–453.415)	128.605 (85.633–476.725)	388.745 (174.268–796.868)
MIP-1β (pg/mL)	9.805 (8.04–12.223)	6.6 (3.865–10.308)	9.57 (6.76–13.948)
PDGF-AA (pg/mL)	1218.17 (936.063–1650.358)	879.905 (668.52–1172.283)	1475.935 (1133.783–1888.875)
PDGF-AB/BB (pg/mL)	3054.77 (2559.113–3722.473)	2472.97 (2080.29–2972.42)	3016.7 (2614.513–3862.918)
TNF-α (pg/mL)	3.54 (1.755–4.975)	1.175 (0.678–2.41)	5.58 (2.86–7.238)
VEGF-A (pg/mL)	54.175 (22.29–78.328)	40.525 (15.195–68.043)	65.375 (37.53–164.385)
IL-18 (pg/mL)	6.21 (3.445–9.308)	3.765 (1.695–5.333)	7.685 (6.518–16.785)

STable 4 legend: Median values and interquartile ranges for measured cyto- and chemokines for each subgroup.

SFig. 1 (a-d): Color coded heatmaps showing correlation coefficients (rho) as the result of spearman-rank tests. If more than 50% of the values for a specific marker were unmeasurable across all groups, the marker was excluded from further analyses. Red indicates a positive correlation, blue indicates a negative correlation, and white indicates no correlation. The intensity of the color represents the strength of the correlation. This figure was created using GraphPad Prism Version 10.3.1.

a) Correlation across all groups.

b) Correlation within the case group.

c) Correlation within the stroke-control group

d) Correlation within the COVID-19 control group

e) Differences between correlation coefficients (Δρ) in the case and stroke control groups. The intensity of the magenta color corresponds to greater distances.

f) Differences between correlation coefficients (Δρ) in the case and COVID-19 control groups. The intensity of the magenta color corresponds to greater distances.

a)

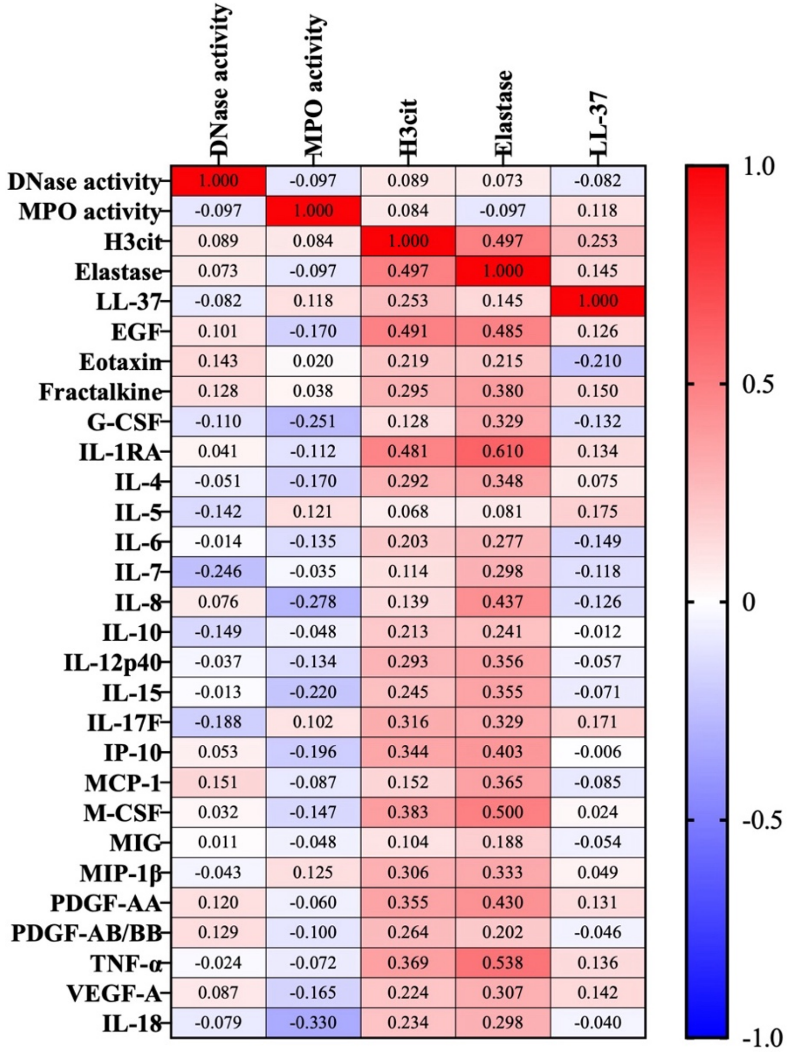
DNase activity
MPO activity
H3cit
Elastase
LL-37
DNase activity 1.000 -0.097 0.089 0.073 -0.082
MPO activity -0.097 1.000 0.084 -0.097 0.118
H3cit 0.089 0.084 1.000 0.497 0.253
Elastase 0.073 -0.097 0.497 1.000 0.145
LL-37 -0.082 0.118 0.253 0.145 1.000
EGF 0.101 -0.170 0.491 0.485 0.126
Eotaxin 0.143 0.020 0.219 0.215 -0.210
Fractalkine 0.128 0.038 0.295 0.380 0.150
G-CSF -0.110 -0.251 0.128 0.329 -0.132
IL-1RA 0.041 -0.112 0.481 0.610 0.134
IL-4 -0.051 -0.170 0.292 0.348 0.075
IL-5 -0.142 0.121 0.068 0.081 0.175
IL-6 -0.014 -0.135 0.203 0.277 -0.149
IL-7 -0.246 -0.035 0.114 0.298 -0.118
IL-8 0.076 -0.278 0.139 0.437 -0.126
IL-10 -0.149 -0.048 0.213 0.241 -0.012
IL-12p40 -0.037 -0.134 0.293 0.356 -0.057
IL-15 -0.013 -0.220 0.245 0.355 -0.071
IL-17F -0.188 0.102 0.316 0.329 0.171
IP-10 0.053 -0.196 0.344 0.403 -0.006
MCP-1 0.151 -0.087 0.152 0.365 -0.085
M-CSF 0.032 -0.147 0.383 0.500 0.024
MIG 0.011 -0.048 0.104 0.188 -0.054
MIP-1β -0.043 0.125 0.306 0.333 0.049
PDGF-AA 0.120 -0.060 0.355 0.430 0.131
PDGF-AB/BB 0.129 -0.100 0.264 0.202 -0.046
TNF-α -0.024 -0.072 0.369 0.538 0.136
VEGF-A 0.087 -0.165 0.224 0.307 0.142
IL-18 -0.079 -0.330 0.234 0.298 -0.040
1.0
0.5
0
-0.5
-1.0

b)

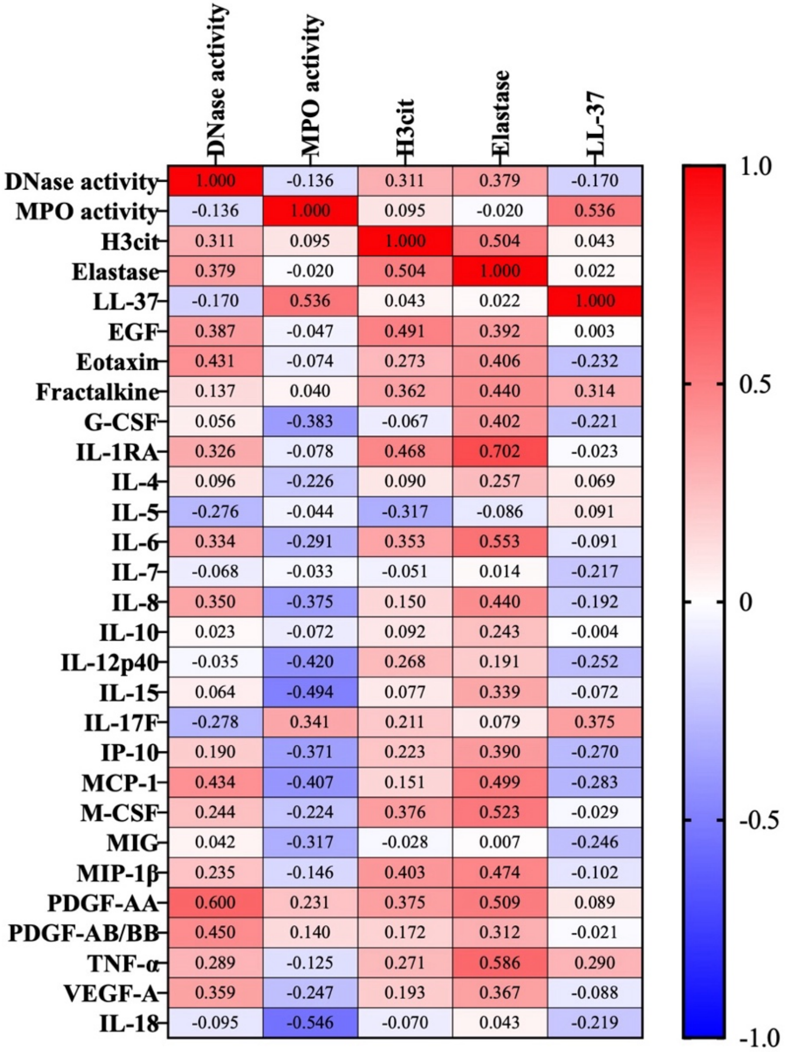

	DNase activity	MPO activity	H3cit	Elastase	LL-37
DNase activity	1.000	-0.136	0.311	0.379	-0.170
MPO activity	-0.136	1.000	0.095	-0.020	0.536
H3cit	0.311	0.095	1.000	0.504	0.043
Elastase	0.379	-0.020	0.504	1.000	0.022
LL-37	-0.170	0.536	0.043	0.022	1.000
EGF	0.387	-0.047	0.491	0.392	0.003
Eotaxin	0.431	-0.074	0.273	0.406	-0.232
Fractalkine	0.137	0.040	0.362	0.440	0.314
G-CSF	0.056	-0.383	-0.067	0.402	-0.221
IL-1RA	0.326	-0.078	0.468	0.702	-0.023
IL-4	0.096	-0.226	0.090	0.257	0.069
IL-5	-0.276	-0.044	-0.317	-0.086	0.091
IL-6	0.334	-0.291	0.353	0.553	-0.091
IL-7	-0.068	-0.033	-0.051	0.014	-0.217
IL-8	0.350	-0.375	0.150	0.440	-0.192
IL-10	0.023	-0.072	0.092	0.243	-0.004
IL-12p40	-0.035	-0.420	0.268	0.191	-0.252
IL-15	0.064	-0.494	0.077	0.339	-0.072
IL-17F	-0.278	0.341	0.211	0.079	0.375
IP-10	0.190	-0.371	0.223	0.390	-0.270
MCP-1	0.434	-0.407	0.151	0.499	-0.283
M-CSF	0.244	-0.224	0.376	0.523	-0.029
MIG	0.042	-0.317	-0.028	0.007	-0.246
MIP-1β	0.235	-0.146	0.403	0.474	-0.102
PDGF-AA	0.600	0.231	0.375	0.509	0.089
PDGF-AB/BB	0.450	0.140	0.172	0.312	-0.021
TNF-α	0.289	-0.125	0.271	0.586	0.290
VEGF-A	0.359	-0.247	0.193	0.367	-0.088
IL-18	-0.095	-0.546	-0.070	0.043	-0.219

c)

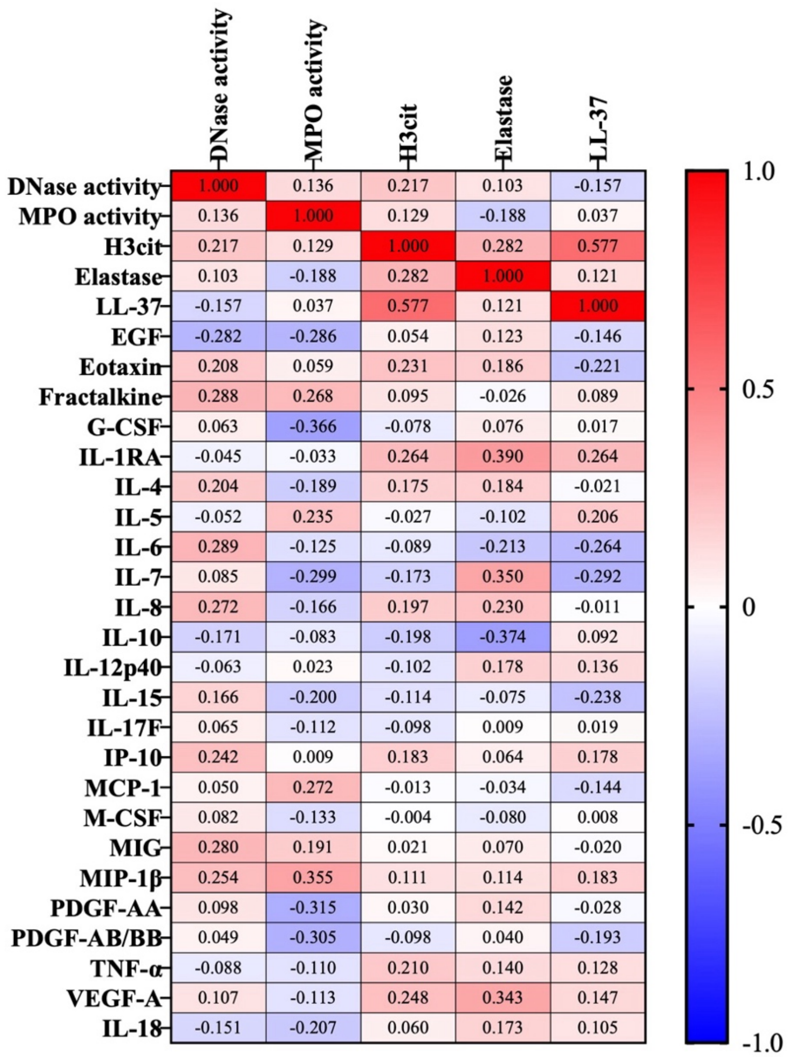
DNase activity
MPO activity
H3cit
Elastase
LL-37
DNase activity 1.000 0.136 0.217 0.103 -0.157
MPO activity 0.136 1.000 0.129 -0.188 0.037
H3cit 0.217 0.129 1.000 0.282 0.577
Elastase 0.103 -0.188 0.282 1.000 0.121
LL-37 -0.157 0.037 0.577 0.121 1.000
EGF -0.282 -0.286 0.054 0.123 -0.146
Eotaxin 0.208 0.059 0.231 0.186 -0.221
Fractalkine 0.288 0.268 0.095 -0.026 0.089
G-CSF 0.063 -0.366 -0.078 0.076 0.017
IL-1RA -0.045 -0.033 0.264 0.390 0.264
IL-4 0.204 -0.189 0.175 0.184 -0.021
IL-5 -0.052 0.235 -0.027 -0.102 0.206
IL-6 0.289 -0.125 -0.089 -0.213 -0.264
IL-7 0.085 -0.299 -0.173 0.350 -0.292
IL-8 0.272 -0.166 0.197 0.230 -0.011
IL-10 -0.171 -0.083 -0.198 -0.374 0.092
IL-12p40 -0.063 0.023 -0.102 0.178 0.136
IL-15 0.166 -0.200 -0.114 -0.075 -0.238
IL-17F 0.065 -0.112 -0.098 0.009 0.019
IP-10 0.242 0.009 0.183 0.064 0.178
MCP-1 0.050 0.272 -0.013 -0.034 -0.144
M-CSF 0.082 -0.133 -0.004 -0.080 0.008
MIG 0.280 0.191 0.021 0.070 -0.020
MIP-1β 0.254 0.355 0.111 0.114 0.183
PDGF-AA 0.098 -0.315 0.030 0.142 -0.028
PDGF-AB/BB 0.049 -0.305 -0.098 0.040 -0.193
TNF-α -0.088 -0.110 0.210 0.140 0.128
VEGF-A 0.107 -0.113 0.248 0.343 0.147
IL-18 -0.151 -0.207 0.060 0.173 0.105
1.0
0.5
0
-0.5
-1.0

d)

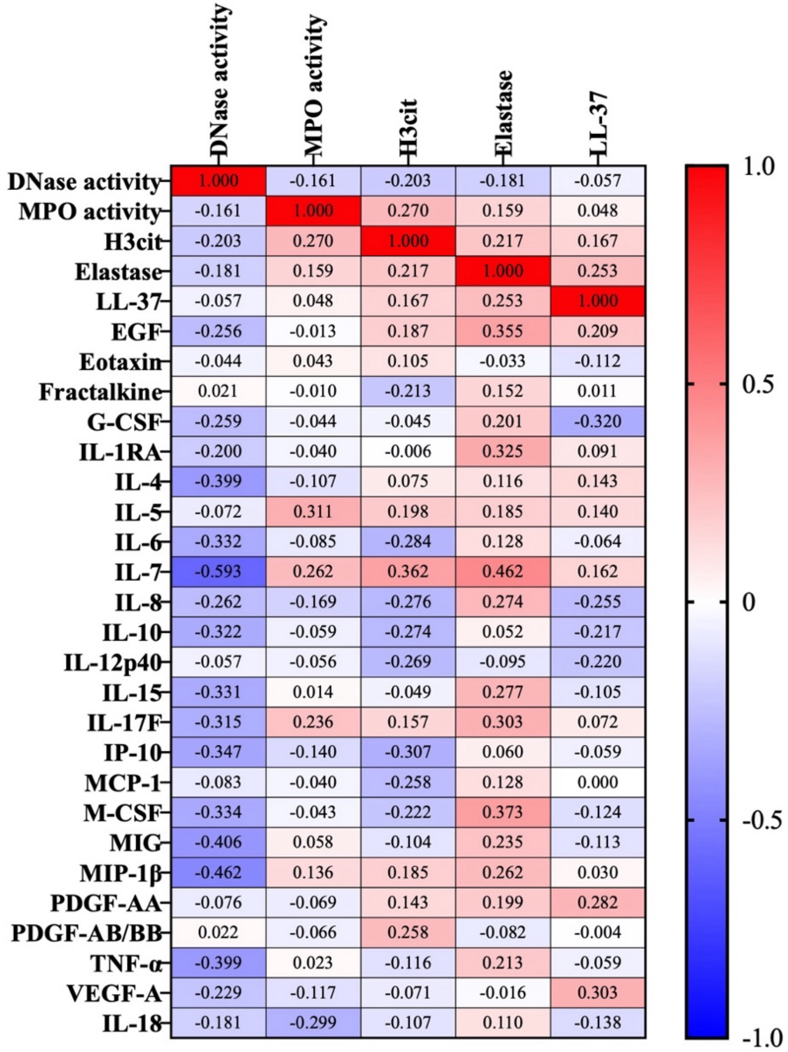
DNase activity
MPO activity
H3cit
Elastase
LL-37
DNase activity 1.000 -0.161 -0.203 -0.181 -0.057
MPO activity -0.161 1.000 0.270 0.159 0.048
H3cit -0.203 0.270 1.000 0.217 0.167
Elastase -0.181 0.159 0.217 1.000 0.253
LL-37 -0.057 0.048 0.167 0.253 1.000
EGF -0.256 -0.013 0.187 0.355 0.209
Eotaxin -0.044 0.043 0.105 -0.033 -0.112
Fractalkine 0.021 -0.010 -0.213 0.152 0.011
G-CSF -0.259 -0.044 -0.045 0.201 -0.320
IL-1RA -0.200 -0.040 -0.006 0.325 0.091
IL-4 -0.399 -0.107 0.075 0.116 0.143
IL-5 -0.072 0.311 0.198 0.185 0.140
IL-6 -0.332 -0.085 -0.284 0.128 -0.064
IL-7 -0.593 0.262 0.362 0.462 0.162
IL-8 -0.262 -0.169 -0.276 0.274 -0.255
IL-10 -0.322 -0.059 -0.274 0.052 -0.217
IL-12p40 -0.057 -0.056 -0.269 -0.095 -0.220
IL-15 -0.331 0.014 -0.049 0.277 -0.105
IL-17F -0.315 0.236 0.157 0.303 0.072
IP-10 -0.347 -0.140 -0.307 0.060 -0.059
MCP-1 -0.083 -0.040 -0.258 0.128 0.000
M-CSF -0.334 -0.043 -0.222 0.373 -0.124
MIG -0.406 0.058 -0.104 0.235 -0.113
MIP-1β -0.462 0.136 0.185 0.262 0.030
PDGF-AA -0.076 -0.069 0.143 0.199 0.282
PDGF-AB/BB 0.022 -0.066 0.258 -0.082 -0.004
TNF-α -0.399 0.023 -0.116 0.213 -0.059
VEGF-A -0.229 -0.117 -0.071 -0.016 0.303
IL-18 -0.181 -0.299 -0.107 0.110 -0.138
1.0
0.5
0
-0.5
-1.0

e)

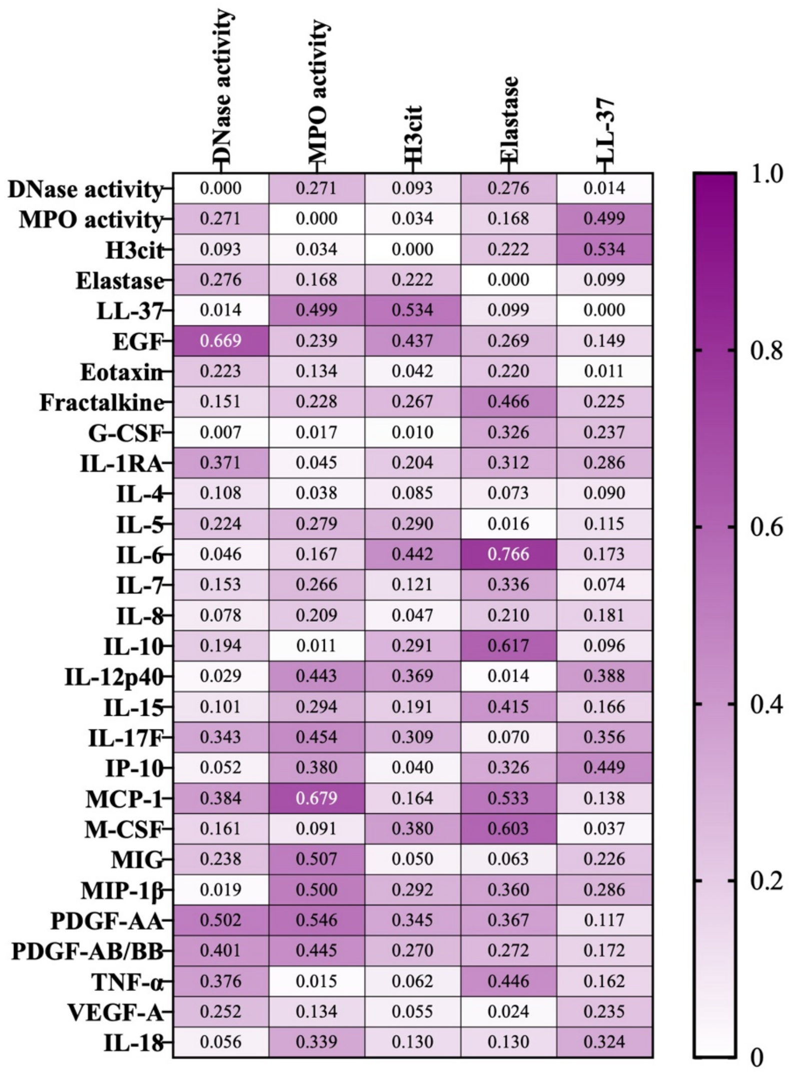
DNase activity
MPO activity
H3cit
Elastase
LL-37
DNase activity 0.000 0.271 0.093 0.276 0.014
MPO activity 0.271 0.000 0.034 0.168 0.499
H3cit 0.093 0.034 0.000 0.222 0.534
Elastase 0.276 0.168 0.222 0.000 0.099
LL-37 0.014 0.499 0.534 0.099 0.000
EGF 0.669 0.239 0.437 0.269 0.149
Eotaxin 0.223 0.134 0.042 0.220 0.011
Fractalkine 0.151 0.228 0.267 0.466 0.225
G-CSF 0.007 0.017 0.010 0.326 0.237
IL-1RA 0.371 0.045 0.204 0.312 0.286
IL-4 0.108 0.038 0.085 0.073 0.090
IL-5 0.224 0.279 0.290 0.016 0.115
IL-6 0.046 0.167 0.442 0.766 0.173
IL-7 0.153 0.266 0.121 0.336 0.074
IL-8 0.078 0.209 0.047 0.210 0.181
IL-10 0.194 0.011 0.291 0.617 0.096
IL-12p40 0.029 0.443 0.369 0.014 0.388
IL-15 0.101 0.294 0.191 0.415 0.166
IL-17F 0.343 0.454 0.309 0.070 0.356
IP-10 0.052 0.380 0.040 0.326 0.449
MCP-1 0.384 0.679 0.164 0.533 0.138
M-CSF 0.161 0.091 0.380 0.603 0.037
MIG 0.238 0.507 0.050 0.063 0.226
MIP-1β 0.019 0.500 0.292 0.360 0.286
PDGF-AA 0.502 0.546 0.345 0.367 0.117
PDGF-AB/BB 0.401 0.445 0.270 0.272 0.172
TNF-α 0.376 0.015 0.062 0.446 0.162
VEGF-A 0.252 0.134 0.055 0.024 0.235
IL-18 0.056 0.339 0.130 0.130 0.324
1.0
0.8
0.6
0.4
0.2
0

f)

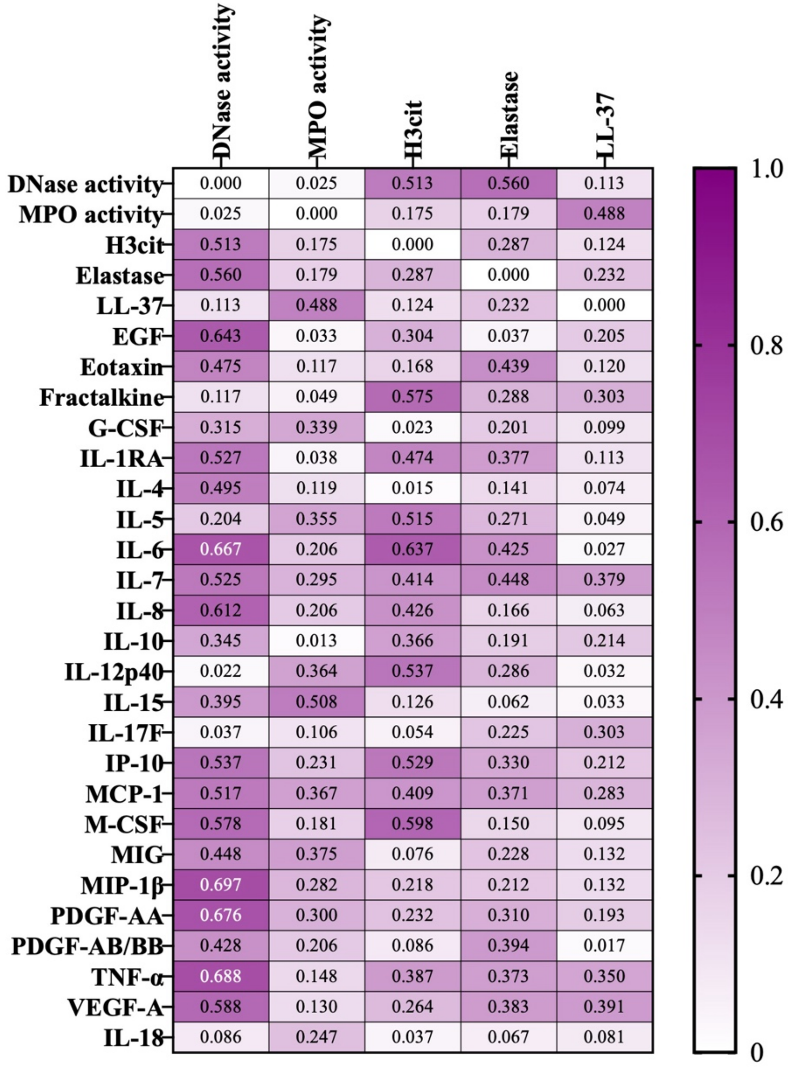
DNase activity
MPO activity
H3cit
Elastase
LL-37
DNase activity 0.000 0.025 0.513 0.560 0.113
MPO activity 0.025 0.000 0.175 0.179 0.488
H3cit 0.513 0.175 0.000 0.287 0.124
Elastase 0.560 0.179 0.287 0.000 0.232
LL-37 0.113 0.488 0.124 0.232 0.000
EGF 0.643 0.033 0.304 0.037 0.205
Eotaxin 0.475 0.117 0.168 0.439 0.120
Fractalkine 0.117 0.049 0.575 0.288 0.303
G-CSF 0.315 0.339 0.023 0.201 0.099
IL-1RA 0.527 0.038 0.474 0.377 0.113
IL-4 0.495 0.119 0.015 0.141 0.074
IL-5 0.204 0.355 0.515 0.271 0.049
IL-6 0.667 0.206 0.637 0.425 0.027
IL-7 0.525 0.295 0.414 0.448 0.379
IL-8 0.612 0.206 0.426 0.166 0.063
IL-10 0.345 0.013 0.366 0.191 0.214
IL-12p40 0.022 0.364 0.537 0.286 0.032
IL-15 0.395 0.508 0.126 0.062 0.033
IL-17F 0.037 0.106 0.054 0.225 0.303
IP-10 0.537 0.231 0.529 0.330 0.212
MCP-1 0.517 0.367 0.409 0.371 0.283
M-CSF 0.578 0.181 0.598 0.150 0.095
MIG 0.448 0.375 0.076 0.228 0.132
MIP-1β 0.697 0.282 0.218 0.212 0.132
PDGF-AA 0.676 0.300 0.232 0.310 0.193
PDGF-AB/BB 0.428 0.206 0.086 0.394 0.017
TNF-α 0.688 0.148 0.387 0.373 0.350
VEGF-A 0.588 0.130 0.264 0.383 0.391
IL-18 0.086 0.247 0.037 0.067 0.081
1.0
0.8
0.6
0.4
0.2
0

SFig. 2 (a-d): NET-NET, NET-Cytokine and Cytokine-Cytokine correlations across all groups (a) or in each subgroup, respectively (b: case group, c: stroke-control group, d: COVID-19 control group). Please note that several cytokines were excluded from further analyses because over 50% were non measurable across all groups.

a)

	DNase activity	MPO activity	H3cit	Elastase	LL-37	EGF	Eotaxin	Fractalkine	G-CSF	IL-1RA	IL-4	IL-5	IL-6	IL-7	IL-8	IL-10	IL-12p40	IL-15	IL-17F	IP-10	MCP-1	M-CSF	MIG	MIP-1β	PDGF-AA	PDGF-AB/BB	TNF-α	VEGF-A	IL-18
DNase activity	1.000	-0.097	0.089	0.073	-0.082	0.101	0.143	0.128	-0.110	0.041	-0.051	-0.142	-0.014	-0.246	0.076	-0.149	-0.037	-0.013	-0.188	0.053	0.151	0.032	0.011	-0.043	0.120	0.129	-0.024	0.087	-0.079
MPO activity	-0.097	1.000	0.084	-0.097	0.118	-0.170	0.020	0.038	-0.251	-0.112	-0.170	0.121	-0.135	-0.035	-0.278	-0.048	-0.134	-0.220	0.102	-0.196	-0.087	-0.147	-0.048	0.125	-0.060	-0.100	-0.072	-0.165	-0.330
H3cit	0.089	0.084	1.000	0.497	0.253	0.491	0.219	0.295	0.128	0.481	0.292	0.068	0.203	0.114	0.139	0.213	0.293	0.245	0.316	0.344	0.152	0.383	0.104	0.306	0.355	0.264	0.369	0.224	0.234
Elastase	0.073	-0.097	0.497	1.000	0.145	0.485	0.215	0.380	0.329	0.610	0.348	0.081	0.277	0.298	0.437	0.241	0.356	0.355	0.329	0.403	0.365	0.500	0.188	0.333	0.430	0.202	0.538	0.307	0.298
LL-37	-0.082	0.118	0.253	0.145	1.000	0.126	-0.210	0.150	-0.132	0.134	0.075	0.175	-0.149	-0.118	-0.126	-0.012	-0.057	-0.071	0.171	-0.006	-0.085	0.024	-0.054	0.049	0.131	-0.046	0.136	0.142	-0.040
EGF	0.101	-0.170	0.491	0.485	0.126	1.000	0.200	0.378	0.333	0.596	0.508	0.092	0.337	0.211	0.228	0.362	0.448	0.506	0.414	0.527	0.359	0.595	0.158	0.229	0.590	0.444	0.552	0.371	0.423
Eotaxin	0.143	0.020	0.219	0.215	-0.210	0.200	1.000	0.236	0.301	0.339	0.259	-0.003	0.333	0.212	0.422	0.050	0.193	0.260	0.110	0.296	0.360	0.272	0.204	0.284	0.251	0.222	0.301	0.071	0.197
Fractalkine	0.128	0.038	0.295	0.380	0.150	0.378	0.236	1.000	0.280	0.544	0.391	0.319	0.386	0.160	0.368	0.433	0.533	0.457	0.439	0.483	0.373	0.614	0.315	0.507	0.338	0.124	0.714	0.142	0.257
G-CSF	-0.110	-0.251	0.128	0.329	-0.132	0.333	0.301	0.280	1.000	0.505	0.512	0.051	0.556	0.271	0.391	0.393	0.439	0.686	0.373	0.441	0.350	0.503	0.214	0.200	0.278	0.145	0.490	0.249	0.409
IL-1RA	0.041	-0.112	0.481	0.610	0.134	0.596	0.339	0.544	0.505	1.000	0.501	0.184	0.642	0.253	0.579	0.585	0.564	0.667	0.508	0.653	0.520	0.779	0.330	0.385	0.448	0.185	0.720	0.252	0.456
IL-4	-0.051	-0.170	0.292	0.348	0.075	0.508	0.259	0.391	0.512	0.501	1.000	0.193	0.396	0.412	0.339	0.339	0.451	0.570	0.478	0.473	0.290	0.515	0.197	0.279	0.518	0.429	0.485	0.637	0.392
IL-5	-0.142	0.121	0.068	0.081	0.175	0.092	-0.003	0.319	0.051	0.184	0.193	1.000	0.153	0.179	0.149	0.215	0.345	0.194	0.332	0.344	0.215	0.339	0.323	0.314	-0.020	-0.013	0.290	-0.033	0.142
IL-6	-0.014	-0.135	0.203	0.277	-0.149	0.337	0.333	0.386	0.556	0.642	0.396	0.153	1.000	0.145	0.639	0.522	0.517	0.686	0.331	0.586	0.478	0.714	0.426	0.253	0.150	0.012	0.594	0.191	0.326
IL-7	-0.246	-0.035	0.114	0.298	-0.118	0.211	0.212	0.160	0.271	0.253	0.412	0.179	0.145	1.000	0.298	0.296	0.249	0.212	0.378	0.117	0.046	0.199	0.045	0.299	0.392	0.316	0.205	0.377	0.239
IL-8	0.076	-0.278	0.139	0.437	-0.126	0.228	0.422	0.368	0.391	0.579	0.339	0.149	0.639	0.298	1.000	0.478	0.411	0.555	0.296	0.580	0.516	0.622	0.472	0.345	0.213	0.108	0.541	0.182	0.361
IL-10	-0.149	-0.048	0.213	0.241	-0.012	0.362	0.050	0.433	0.393	0.585	0.339	0.215	0.522	0.296	0.478	1.000	0.485	0.526	0.411	0.554	0.384	0.608	0.401	0.318	0.259	0.074	0.528	0.118	0.382
IL-12p40	-0.037	-0.134	0.293	0.356	-0.057	0.448	0.193	0.533	0.439	0.564	0.451	0.345	0.517	0.249	0.411	0.485	1.000	0.611	0.590	0.582	0.396	0.646	0.415	0.319	0.310	0.101	0.636	0.252	0.439
IL-15	-0.013	-0.220	0.245	0.355	-0.071	0.506	0.260	0.457	0.686	0.667	0.570	0.194	0.686	0.212	0.555	0.526	0.611	1.000	0.515	0.613	0.451	0.753	0.291	0.223	0.346	0.245	0.673	0.240	0.585
IL-17F	-0.188	0.102	0.316	0.329	0.171	0.414	0.110	0.439	0.373	0.508	0.478	0.332	0.331	0.378	0.296	0.411	0.590	0.515	1.000	0.350	0.184	0.520	0.152	0.274	0.407	0.281	0.512	0.274	0.306
IP-10	0.053	-0.196	0.344	0.403	-0.006	0.527	0.296	0.483	0.441	0.653	0.473	0.344	0.586	0.117	0.580	0.554	0.582	0.613	0.350	1.000	0.582	0.729	0.630	0.384	0.296	0.068	0.667	0.282	0.460
MCP-1	0.151	-0.087	0.152	0.365	-0.085	0.359	0.360	0.373	0.350	0.520	0.290	0.215	0.478	0.046	0.516	0.384	0.396	0.451	0.184	0.582	1.000	0.493	0.415	0.369	0.169	0.158	0.528	0.203	0.400
M-CSF	0.032	-0.147	0.383	0.500	0.024	0.595	0.272	0.614	0.503	0.779	0.515	0.339	0.714	0.199	0.622	0.608	0.646	0.753	0.520	0.729	0.493	1.000	0.442	0.419	0.426	0.180	0.757	0.178	0.443
MIG	0.011	-0.048	0.104	0.188	-0.054	0.158	0.204	0.315	0.214	0.330	0.197	0.323	0.426	0.045	0.472	0.401	0.415	0.291	0.152	0.630	0.415	0.442	1.000	0.440	-0.044	-0.212	0.415	0.123	0.122
MIP-1β	-0.043	0.125	0.306	0.333	0.049	0.229	0.284	0.507	0.200	0.385	0.279	0.314	0.253	0.299	0.345	0.318	0.319	0.223	0.274	0.384	0.369	0.419	0.440	1.000	0.269	0.267	0.396	0.223	0.174
PDGF-AA	0.120	-0.060	0.355	0.430	0.131	0.590	0.251	0.338	0.278	0.448	0.518	-0.020	0.150	0.392	0.213	0.259	0.310	0.346	0.407	0.296	0.169	0.426	-0.044	0.269	1.000	0.697	0.494	0.457	0.308
PDGF-AB/BB	0.129	-0.100	0.264	0.202	-0.046	0.444	0.222	0.124	0.145	0.185	0.429	-0.013	0.012	0.316	0.108	0.074	0.101	0.245	0.281	0.068	0.158	0.180	-0.212	0.267	0.697	1.000	0.197	0.382	0.340
TNF-α	-0.024	-0.072	0.369	0.538	0.136	0.552	0.301	0.714	0.490	0.720	0.485	0.290	0.594	0.205	0.541	0.528	0.636	0.673	0.512	0.667	0.528	0.757	0.415	0.396	0.494	0.197	1.000	0.200	0.500
VEGF-A	0.087	-0.165	0.224	0.307	0.142	0.371	0.071	0.142	0.249	0.252	0.637	-0.033	0.191	0.377	0.182	0.118	0.252	0.240	0.274	0.282	0.203	0.178	0.123	0.223	0.457	0.382	0.200	1.000	0.248
IL-18	-0.079	-0.330	0.234	0.298	-0.040	0.423	0.197	0.257	0.409	0.456	0.392	0.142	0.326	0.239	0.361	0.382	0.439	0.585	0.306	0.460	0.400	0.443	0.122	0.174	0.308	0.340	0.500	0.248	1.000

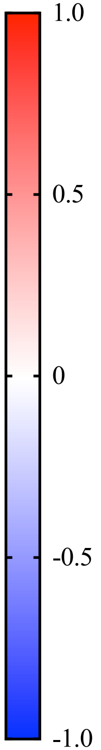

b)

	DNase activity	MPO activity	H3cit	Elastase	LL-37	EGF	Eotaxin	Fractalkine	G-CSF	IL-1RA	IL-4	IL-5	IL-6	IL-7	IL-8	IL-10	IL-12p40	IL-15	IL-17F	IP-10	MCP-1	M-CSF	MIG	MIP-1β	PDGF-AA	PDGF-AB/BB	TNF-α	VEGF-A	IL-18
DNase activity	1.000	-0.136	0.311	0.379	-0.170	0.387	0.431	0.137	0.056	0.326	0.096	-0.276	0.334	-0.068	0.350	0.023	-0.035	0.064	-0.278	0.190	0.434	0.244	0.042	0.235	0.600	0.450	0.289	0.359	-0.095
MPO activity	-0.136	1.000	0.095	-0.020	0.536	-0.047	-0.074	0.040	-0.383	-0.078	-0.226	-0.044	-0.291	-0.033	-0.375	-0.072	-0.420	-0.494	0.341	-0.371	-0.407	-0.224	-0.317	-0.146	0.231	0.140	-0.125	-0.247	-0.546
H3cit	0.311	0.095	1.000	0.504	0.043	0.491	0.273	0.362	-0.067	0.468	0.090	-0.317	0.353	-0.051	0.150	0.092	0.268	0.077	0.211	0.223	0.151	0.376	-0.028	0.403	0.375	0.172	0.271	0.193	-0.070
Elastase	0.379	-0.020	0.504	1.000	0.022	0.392	0.406	0.440	0.402	0.702	0.257	-0.086	0.553	0.014	0.440	0.243	0.191	0.339	0.079	0.390	0.499	0.523	0.007	0.474	0.509	0.312	0.586	0.367	0.043
LL-37	-0.170	0.536	0.043	0.022	1.000	0.003	-0.232	0.314	-0.221	-0.023	0.069	0.091	-0.091	-0.217	-0.192	-0.004	-0.252	-0.072	0.375	-0.270	-0.283	-0.029	-0.246	-0.102	0.089	-0.021	0.290	-0.088	-0.219
EGF	0.387	-0.047	0.491	0.392	0.003	1.000	0.171	0.260	0.180	0.506	0.271	-0.364	0.529	-0.217	-0.094	-0.042	0.095	0.349	0.149	0.111	0.232	0.414	-0.332	-0.031	0.373	0.337	0.324	0.051	0.036
Eotaxin	0.431	-0.074	0.273	0.406	-0.232	0.171	1.000	0.018	0.346	0.430	0.175	-0.075	0.332	0.060	0.503	0.087	0.078	0.050	-0.278	0.389	0.572	0.090	0.292	0.356	0.307	0.162	0.190	0.190	0.150
Fractalkine	0.137	0.040	0.362	0.440	0.314	0.260	0.018	1.000	0.227	0.469	0.367	0.104	0.319	0.040	0.136	0.295	0.273	0.404	0.178	0.129	0.236	0.464	0.002	0.250	0.297	0.144	0.684	0.122	-0.141
G-CSF	0.056	-0.383	-0.067	0.402	-0.221	0.180	0.346	0.227	1.000	0.399	0.507	-0.054	0.556	0.231	0.375	0.289	0.215	0.583	-0.097	0.180	0.433	0.323	0.012	0.115	0.026	0.079	0.415	0.306	0.190
IL-1RA	0.326	-0.078	0.468	0.702	-0.023	0.506	0.430	0.469	0.399	1.000	0.238	-0.118	0.753	-0.057	0.493	0.433	0.197	0.498	0.038	0.531	0.492	0.740	0.041	0.276	0.289	0.052	0.646	0.009	0.084
IL-4	0.096	-0.226	0.090	0.257	0.069	0.271	0.175	0.367	0.507	0.238	1.000	-0.132	0.473	0.040	0.105	0.111	0.406	0.614	0.167	0.228	0.301	0.370	0.016	0.181	0.371	0.378	0.369	0.587	-0.038
IL-5	-0.276	-0.044	-0.317	-0.086	0.091	-0.364	-0.075	0.104	-0.054	-0.118	-0.132	1.000	-0.274	0.308	-0.015	0.186	0.154	-0.013	0.037	0.092	-0.035	-0.072	0.185	0.005	-0.176	-0.216	-0.047	-0.348	0.080
IL-6	0.334	-0.291	0.353	0.553	-0.091	0.529	0.332	0.319	0.556	0.753	0.473	-0.274	1.000	-0.139	0.469	0.396	0.412	0.728	0.091	0.383	0.495	0.780	0.158	0.185	0.135	0.018	0.615	0.339	0.208
IL-7	-0.068	-0.033	-0.051	0.014	-0.217	-0.217	0.060	0.040	0.231	-0.057	0.040	0.308	-0.139	1.000	-0.098	0.191	0.185	-0.169	0.061	-0.265	-0.049	-0.229	-0.128	0.374	0.169	0.238	-0.259	0.139	0.074
IL-8	0.350	-0.375	0.150	0.440	-0.192	-0.094	0.503	0.136	0.375	0.493	0.105	-0.015	0.469	-0.098	1.000	0.515	0.273	0.307	-0.154	0.415	0.529	0.418	0.618	0.473	-0.060	-0.217	0.405	0.143	0.024
IL-10	0.023	-0.072	0.092	0.243	-0.004	-0.042	0.087	0.295	0.289	0.433	0.111	0.186	0.396	0.191	0.515	1.000	0.155	0.375	0.268	0.196	0.186	0.401	0.311	0.341	-0.044	0.051	0.268	-0.037	0.015
IL-12p40	-0.035	-0.420	0.268	0.191	-0.252	0.095	0.078	0.273	0.215	0.197	0.406	0.154	0.412	0.185	0.273	0.155	1.000	0.461	0.249	0.262	0.186	0.452	0.324	0.284	-0.016	-0.160	0.222	0.218	0.246
IL-15	0.064	-0.494	0.077	0.339	-0.072	0.349	0.050	0.404	0.583	0.498	0.614	-0.013	0.728	-0.169	0.307	0.375	0.461	1.000	0.103	0.405	0.349	0.682	0.164	0.000	-0.020	0.075	0.611	0.184	0.425
IL-17F	-0.278	0.341	0.211	0.079	0.375	0.149	-0.278	0.178	-0.097	0.038	0.167	0.037	0.091	0.061	-0.154	0.268	0.249	0.103	1.000	-0.362	-0.453	0.144	-0.303	-0.124	0.032	0.072	-0.023	-0.080	-0.145
IP-10	0.190	-0.371	0.223	0.390	-0.270	0.111	0.389	0.129	0.180	0.531	0.228	0.092	0.383	-0.265	0.415	0.196	0.262	0.405	-0.362	1.000	0.660	0.547	0.481	0.229	0.136	-0.066	0.500	0.030	0.241
MCP-1	0.434	-0.407	0.151	0.499	-0.283	0.232	0.572	0.236	0.433	0.492	0.301	-0.035	0.495	-0.049	0.529	0.186	0.186	0.349	-0.453	0.660	1.000	0.279	0.365	0.346	0.180	0.103	0.492	0.371	0.101
M-CSF	0.244	-0.224	0.376	0.523	-0.029	0.414	0.090	0.464	0.323	0.740	0.370	-0.072	0.780	-0.229	0.418	0.401	0.452	0.682	0.144	0.547	0.279	1.000	0.261	0.183	0.196	-0.085	0.664	0.016	0.039
MIG	0.042	-0.317	-0.028	0.007	-0.246	-0.332	0.292	0.002	0.012	0.041	0.016	0.185	0.158	-0.128	0.618	0.311	0.324	0.164	-0.303	0.481	0.365	0.261	1.000	0.417	-0.264	-0.296	0.248	0.012	0.097
MIP-1β	0.235	-0.146	0.403	0.474	-0.102	-0.031	0.356	0.250	0.115	0.276	0.181	0.005	0.185	0.374	0.473	0.341	0.284	0.000	-0.124	0.229	0.346	0.183	0.417	1.000	0.256	0.230	0.171	0.440	-0.025
PDGF-AA	0.600	0.231	0.375	0.509	0.089	0.373	0.307	0.297	0.026	0.289	0.371	-0.176	0.135	0.169	-0.060	-0.044	-0.016	-0.020	0.032	0.136	0.180	0.196	-0.264	0.256	1.000	0.711	0.228	0.385	-0.209
PDGF-AB/BB	0.450	0.140	0.172	0.312	-0.021	0.337	0.162	0.144	0.079	0.052	0.378	-0.216	0.018	0.238	-0.217	0.051	-0.160	0.075	0.072	-0.066	0.103	-0.085	-0.296	0.230	0.711	1.000	0.029	0.459	-0.035
TNF-α	0.289	-0.125	0.271	0.586	0.290	0.324	0.190	0.684	0.415	0.646	0.369	-0.047	0.615	-0.259	0.405	0.268	0.222	0.611	-0.023	0.500	0.492	0.664	0.248	0.171	0.228	0.029	1.000	0.152	0.140
VEGF-A	0.359	-0.247	0.193	0.367	-0.088	0.051	0.190	0.122	0.306	0.009	0.587	-0.348	0.339	0.139	0.143	-0.037	0.218	0.184	-0.080	0.030	0.371	0.016	0.012	0.440	0.385	0.459	0.152	1.000	0.017
IL-18	-0.095	-0.546	-0.070	0.043	-0.219	0.036	0.150	-0.141	0.190	0.084	-0.038	0.080	0.208	0.074	0.024	0.015	0.246	0.425	-0.145	0.241	0.101	0.039	0.097	-0.025	-0.209	-0.035	0.140	0.017	1.000

1.0
0.5
0
-0.5
-1.0

c)

	DNase activity	MPO activity	H3cit	Elastase	LL-37	EGF	Eotaxin	Fractalkine	G-CSF	IL-1RA	IL-4	IL-5	IL-6	IL-7	IL-8	IL-10	IL-12p40	IL-15	IL-17F	IP-10	MCP-1	M-CSF	MIG	MIP-1β	PDGF-AA	PDGF-AB/BB	TNF-α	VEGF-A	IL-18
DNase activity	1.000	0.136	0.217	0.103	-0.157	-0.282	0.208	0.288	0.063	-0.045	0.204	-0.052	0.289	0.085	0.272	-0.171	-0.063	0.166	0.065	0.242	0.050	0.082	0.280	0.254	0.098	0.049	-0.088	0.107	-0.151
MPO activity	0.136	1.000	0.129	-0.188	0.037	-0.286	0.059	0.268	-0.366	-0.033	-0.189	0.235	-0.125	-0.299	-0.166	-0.083	0.023	-0.200	-0.112	0.009	0.272	-0.133	0.191	0.355	-0.315	-0.305	-0.110	-0.113	-0.207
H3cit	0.217	0.129	1.000	0.282	0.577	0.054	0.231	0.095	-0.078	0.264	0.175	-0.027	-0.089	-0.173	0.197	-0.198	-0.102	-0.114	-0.098	0.183	-0.013	-0.004	0.021	0.111	0.030	-0.098	0.210	0.248	0.060
Elastase	0.103	-0.188	0.282	1.000	0.121	0.123	0.186	-0.026	0.076	0.390	0.184	-0.102	-0.213	0.350	0.230	-0.374	0.178	-0.075	0.009	0.064	-0.034	-0.080	0.070	0.114	0.142	0.040	0.140	0.343	0.173
LL-37	-0.157	0.037	0.577	0.121	1.000	-0.146	-0.221	0.089	0.017	0.264	-0.021	0.206	-0.264	-0.292	-0.011	0.092	0.136	-0.238	0.019	0.178	-0.144	0.008	-0.020	0.183	-0.028	-0.193	0.128	0.147	0.105
EGF	-0.282	-0.286	0.054	0.123	-0.146	1.000	0.344	0.081	0.272	0.387	0.431	0.022	0.096	0.125	0.020	-0.016	0.175	0.329	0.235	-0.104	0.138	0.224	0.098	0.113	0.434	0.481	0.286	0.365	0.152
Eotaxin	0.208	0.059	0.231	0.186	-0.221	0.344	1.000	0.115	0.224	0.176	0.315	0.046	0.284	0.297	0.486	-0.395	-0.002	0.406	0.167	0.049	0.369	0.338	0.026	0.088	0.135	0.218	0.234	0.054	0.016
Fractalkine	0.288	0.268	0.095	-0.026	0.089	0.081	0.115	1.000	-0.039	0.191	0.285	0.395	0.229	0.092	0.163	0.136	0.222	0.181	0.341	0.205	0.105	0.433	0.096	0.697	0.389	0.179	0.456	0.072	0.114
G-CSF	0.063	-0.366	-0.078	0.076	0.017	0.272	0.224	-0.039	1.000	0.291	0.617	0.225	0.380	0.008	0.109	-0.073	0.227	0.569	0.340	0.175	-0.133	0.384	0.228	0.002	0.319	0.236	0.295	0.358	0.186
IL-1RA	-0.045	-0.033	0.264	0.390	0.264	0.387	0.176	0.191	0.291	1.000	0.567	0.233	0.237	0.307	0.224	0.163	0.465	0.367	0.398	0.023	0.020	0.299	0.129	0.177	0.367	0.177	0.465	0.517	0.384
IL-4	0.204	-0.189	0.175	0.184	-0.021	0.431	0.315	0.285	0.617	0.567	1.000	0.307	0.369	0.207	0.140	-0.052	0.218	0.353	0.364	0.173	0.101	0.394	0.223	0.244	0.441	0.388	0.524	0.566	0.409
IL-5	-0.052	0.235	-0.027	-0.102	0.206	0.022	0.046	0.395	0.225	0.233	0.307	1.000	0.296	0.125	0.218	0.040	0.281	0.336	0.378	0.403	0.334	0.502	0.171	0.650	-0.014	0.236	0.245	0.205	0.082
IL-6	0.289	-0.125	-0.089	-0.213	-0.264	0.096	0.284	0.229	0.380	0.237	0.369	0.296	1.000	0.214	0.533	0.367	0.078	0.588	0.321	0.255	0.105	0.516	0.215	-0.019	0.088	0.127	0.239	0.234	-0.061
IL-7	0.085	-0.299	-0.173	0.350	-0.292	0.125	0.297	0.092	0.008	0.307	0.207	0.125	0.214	1.000	0.489	0.069	0.183	0.193	0.322	-0.105	-0.012	0.300	-0.185	0.059	0.243	0.213	0.146	0.271	0.002
IL-8	0.272	-0.166	0.197	0.230	-0.011	0.020	0.486	0.163	0.109	0.224	0.140	0.218	0.533	0.489	1.000	0.089	0.010	0.460	0.166	0.346	0.105	0.571	0.078	0.024	0.300	0.251	0.298	0.155	0.042
IL-10	-0.171	-0.083	-0.198	-0.374	0.092	-0.016	-0.395	0.136	-0.073	0.163	-0.052	0.040	0.367	0.069	0.089	1.000	0.260	0.034	0.014	-0.151	-0.120	0.344	0.083	-0.156	0.044	-0.128	0.081	-0.021	-0.053
IL-12p40	-0.063	0.023	-0.102	0.178	0.136	0.175	-0.002	0.222	0.227	0.465	0.218	0.281	0.078	0.183	0.010	0.260	1.000	0.316	0.617	-0.238	0.102	0.258	-0.028	0.122	0.292	0.316	0.362	0.298	0.240
IL-15	0.166	-0.200	-0.114	-0.075	-0.238	0.329	0.406	0.181	0.569	0.367	0.353	0.336	0.588	0.193	0.460	0.034	0.316	1.000	0.396	0.153	0.135	0.613	0.093	0.093	0.484	0.482	0.315	0.175	0.185
IL-17F	0.065	-0.112	-0.098	0.009	0.019	0.235	0.167	0.341	0.340	0.398	0.364	0.378	0.321	0.322	0.166	0.014	0.617	0.396	1.000	0.072	0.139	0.253	-0.048	0.241	0.333	0.480	0.311	0.504	0.164
IP-10	0.242	0.009	0.183	0.064	0.178	-0.104	0.049	0.205	0.175	0.023	0.173	0.403	0.255	-0.105	0.346	-0.151	-0.238	0.153	0.072	1.000	0.015	0.208	0.578	0.395	-0.077	-0.112	0.036	0.270	-0.226
MCP-1	0.050	0.272	-0.013	-0.034	-0.144	0.138	0.369	0.105	-0.133	0.020	0.101	0.334	0.105	-0.012	0.105	-0.120	0.102	0.135	0.139	0.015	1.000	0.373	0.126	0.372	0.011	0.377	0.049	0.051	0.189
M-CSF	0.082	-0.133	-0.004	-0.080	0.008	0.224	0.338	0.433	0.384	0.299	0.394	0.502	0.516	0.300	0.571	0.344	0.258	0.613	0.253	0.208	0.373	1.000	0.194	0.385	0.527	0.442	0.529	0.043	0.204
MIG	0.280	0.191	0.021	0.070	-0.020	0.098	0.026	0.096	0.228	0.129	0.223	0.171	0.215	-0.185	0.078	0.083	-0.028	0.093	-0.048	0.578	0.126	0.194	1.000	0.320	-0.122	-0.230	-0.133	0.256	-0.439
MIP-1β	0.254	0.355	0.111	0.114	0.183	0.113	0.088	0.697	0.002	0.177	0.244	0.650	-0.019	0.059	0.024	-0.156	0.122	0.093	0.241	0.395	0.372	0.385	0.320	1.000	0.195	0.195	0.192	0.148	-0.087
PDGF-AA	0.098	-0.315	0.030	0.142	-0.028	0.434	0.135	0.389	0.319	0.367	0.441	-0.014	0.088	0.243	0.300	0.044	0.292	0.484	0.333	-0.077	0.011	0.527	-0.122	0.195	1.000	0.639	0.637	0.190	0.435
PDGF-AB/BB	0.049	-0.305	-0.098	0.040	-0.193	0.481	0.218	0.179	0.236	0.177	0.388	0.236	0.127	0.213	0.251	-0.128	0.316	0.482	0.480	-0.112	0.377	0.442	-0.230	0.195	0.639	1.000	0.316	0.246	0.540
TNF-α	-0.088	-0.110	0.210	0.140	0.128	0.286	0.234	0.456	0.295	0.465	0.524	0.245	0.239	0.146	0.298	0.081	0.362	0.315	0.311	0.036	0.049	0.529	-0.133	0.192	0.637	0.316	1.000	0.065	0.510
VEGF-A	0.107	-0.113	0.248	0.343	0.147	0.365	0.054	0.072	0.358	0.517	0.566	0.205	0.234	0.271	0.155	-0.021	0.298	0.175	0.504	0.270	0.051	0.043	0.256	0.148	0.190	0.246	0.065	1.000	0.109
IL-18	-0.151	-0.207	0.060	0.173	0.105	0.152	0.016	0.114	0.186	0.384	0.409	0.082	-0.061	0.002	0.042	-0.053	0.240	0.185	0.164	-0.226	0.189	0.204	-0.439	-0.087	0.435	0.540	0.510	0.109	1.000

1.0
0.5
0
-0.5
-1.0

d)

	DNase activity	MPO activity	H3cit	Elastase	LL-37	EGF	Eotaxin	Fractalkine	G-CSF	IL-1RA	IL-4	IL-5	IL-6	IL-7	IL-8	IL-10	IL-12p40	IL-15	IL-17F	IP-10	MCP-1	M-CSF	MIG	MIP-1β	PDGF-AA	PDGF-AB/BB	TNF-α	VEGF-A	IL-18
DNase activity	1.000	-0.161	-0.203	-0.181	-0.057	-0.256	-0.044	0.021	-0.259	-0.200	-0.399	-0.072	-0.332	-0.593	-0.262	-0.322	-0.057	-0.331	-0.315	-0.347	-0.083	-0.334	-0.406	-0.462	-0.076	0.022	-0.399	-0.229	-0.181
MPO activity	-0.161	1.000	0.270	0.159	0.048	-0.013	0.043	-0.010	-0.044	-0.040	-0.107	0.311	-0.085	0.262	-0.169	-0.059	-0.056	0.014	0.236	-0.140	-0.040	-0.043	0.058	0.136	-0.069	-0.066	0.023	-0.117	-0.299
H3cit	-0.203	0.270	1.000	0.217	0.167	0.187	0.105	-0.213	-0.045	-0.006	0.075	0.198	-0.284	0.362	-0.276	-0.274	-0.269	-0.049	0.157	-0.307	-0.258	-0.222	-0.104	0.185	0.143	0.258	-0.116	-0.071	-0.107
Elastase	-0.181	0.159	0.217	1.000	0.253	0.355	-0.033	0.152	0.201	0.325	0.116	0.185	0.128	0.462	0.274	0.052	-0.095	0.277	0.303	0.060	0.128	0.373	0.235	0.262	0.199	-0.082	0.213	-0.016	0.110
LL-37	-0.057	0.048	0.167	0.253	1.000	0.209	-0.112	0.011	-0.320	0.091	0.143	0.140	-0.064	0.162	-0.255	-0.217	-0.220	-0.105	0.072	-0.059	0.000	-0.124	-0.113	0.030	0.282	-0.004	-0.059	0.303	-0.138
EGF	-0.256	-0.013	0.187	0.355	0.209	1.000	0.137	-0.105	0.154	0.045	0.432	-0.126	0.077	0.597	0.168	0.160	-0.067	0.012	0.147	0.125	-0.042	0.277	0.058	0.159	0.674	0.395	0.192	0.484	0.154
Eotaxin	-0.044	0.043	0.105	-0.033	-0.112	0.137	1.000	0.577	0.287	0.308	0.180	-0.135	0.118	0.207	0.334	0.211	0.204	0.265	0.239	0.235	0.103	0.210	0.235	0.390	0.291	0.236	0.382	-0.046	0.365
Fractalkine	0.021	-0.010	-0.213	0.152	0.011	-0.105	0.577	1.000	0.200	0.576	0.194	0.249	0.369	0.143	0.494	0.387	0.572	0.319	0.342	0.621	0.489	0.528	0.628	0.351	-0.018	-0.343	0.661	-0.046	0.393
G-CSF	-0.259	-0.044	-0.045	0.201	-0.320	0.154	0.287	0.200	1.000	0.461	0.121	-0.388	0.543	0.218	0.440	0.521	0.364	0.604	0.243	0.342	0.359	0.422	0.317	0.147	-0.037	-0.192	0.406	-0.084	0.328
IL-1RA	-0.200	-0.040	-0.006	0.325	0.091	0.045	0.308	0.576	0.461	1.000	0.362	0.034	0.621	0.352	0.710	0.567	0.447	0.622	0.589	0.514	0.473	0.679	0.528	0.324	0.094	-0.279	0.631	0.042	0.392
IL-4	-0.399	-0.107	0.075	0.116	0.143	0.432	0.180	0.194	0.121	0.362	1.000	-0.087	0.052	0.651	0.271	0.330	0.065	0.276	0.434	0.258	0.058	0.237	0.105	0.101	0.431	0.164	0.183	0.660	0.317
IL-5	-0.072	0.311	0.198	0.185	0.140	-0.126	-0.135	0.249	-0.388	0.034	-0.087	1.000	0.044	-0.003	-0.101	-0.159	0.156	-0.163	0.294	0.054	-0.021	0.157	0.338	-0.083	-0.278	-0.376	0.193	-0.313	-0.281
IL-6	-0.332	-0.085	-0.284	0.128	-0.064	0.077	0.118	0.369	0.543	0.621	0.052	0.044	1.000	0.173	0.711	0.655	0.569	0.589	0.281	0.686	0.595	0.727	0.674	0.136	-0.281	-0.498	0.687	-0.064	0.367
IL-7	-0.593	0.262	0.362	0.462	0.162	0.597	0.207	0.143	0.218	0.352	0.651	-0.003	0.173	1.000	0.356	0.281	0.016	0.336	0.453	0.234	0.014	0.326	0.268	0.415	0.473	0.276	0.345	0.544	0.236
IL-8	-0.262	-0.169	-0.276	0.274	-0.255	0.168	0.334	0.494	0.440	0.710	0.271	-0.101	0.711	0.356	1.000	0.648	0.404	0.626	0.392	0.695	0.551	0.738	0.667	0.364	0.080	-0.060	0.692	0.040	0.609
IL-10	-0.322	-0.059	-0.274	0.052	-0.217	0.160	0.211	0.387	0.521	0.567	0.330	-0.159	0.655	0.281	0.648	1.000	0.567	0.565	0.300	0.805	0.578	0.510	0.655	0.180	-0.017	-0.349	0.664	0.175	0.563
IL-12p40	-0.057	-0.056	-0.269	-0.095	-0.220	-0.067	0.204	0.572	0.364	0.447	0.065	0.156	0.569	0.016	0.404	0.567	1.000	0.471	0.365	0.666	0.484	0.424	0.629	0.041	-0.247	-0.481	0.691	-0.117	0.175
IL-15	-0.331	0.014	-0.049	0.277	-0.105	0.012	0.265	0.319	0.604	0.622	0.276	-0.163	0.589	0.336	0.626	0.565	0.471	1.000	0.696	0.429	0.462	0.462	0.415	0.152	-0.052	-0.196	0.611	0.047	0.597
IL-17F	-0.315	0.236	0.157	0.303	0.072	0.147	0.239	0.342	0.243	0.589	0.434	0.294	0.281	0.453	0.392	0.300	0.365	0.696	1.000	0.202	0.330	0.426	0.392	0.233	0.125	-0.007	0.517	0.083	0.320
IP-10	-0.347	-0.140	-0.307	0.060	-0.059	0.125	0.235	0.621	0.342	0.514	0.258	0.054	0.686	0.234	0.695	0.805	0.666	0.429	0.202	1.000	0.655	0.599	0.807	0.258	-0.051	-0.400	0.795	0.107	0.500
MCP-1	-0.083	-0.040	-0.258	0.128	0.000	-0.042	0.103	0.489	0.359	0.473	0.058	-0.021	0.595	0.014	0.551	0.578	0.484	0.462	0.330	0.655	1.000	0.432	0.677	0.156	-0.107	-0.283	0.629	-0.040	0.537
M-CSF	-0.334	-0.043	-0.222	0.373	-0.124	0.277	0.210	0.528	0.422	0.679	0.237	0.157	0.727	0.326	0.738	0.510	0.424	0.462	0.426	0.599	0.432	1.000	0.673	0.380	-0.023	-0.318	0.659	0.015	0.346
MIG	-0.406	0.058	-0.104	0.235	-0.113	0.058	0.235	0.628	0.317	0.528	0.105	0.338	0.674	0.268	0.667	0.655	0.629	0.415	0.392	0.807	0.677	0.673	1.000	0.478	-0.193	-0.375	0.815	-0.149	0.370
MIP-1β	-0.462	0.136	0.185	0.262	0.030	0.159	0.390	0.351	0.147	0.324	0.101	-0.083	0.136	0.415	0.364	0.180	0.041	0.152	0.233	0.258	0.156	0.380	0.478	1.000	0.096	0.211	0.374	0.010	0.204
PDGF-AA	-0.076	-0.069	0.143	0.199	0.282	0.674	0.291	-0.018	-0.037	0.094	0.431	-0.278	-0.281	0.473	0.080	-0.017	-0.247	-0.052	0.125	-0.051	-0.107	-0.023	-0.193	0.096	1.000	0.656	0.052	0.562	0.194
PDGF-AB/BB	0.022	-0.066	0.258	-0.082	-0.004	0.395	0.236	-0.343	-0.192	-0.279	0.164	-0.376	-0.498	0.276	-0.060	-0.349	-0.481	-0.196	-0.007	-0.400	-0.283	-0.318	-0.375	0.211	0.656	1.000	-0.287	0.263	0.067
TNF-α	-0.399	0.023	-0.116	0.213	-0.059	0.192	0.382	0.661	0.406	0.631	0.183	0.193	0.687	0.345	0.692	0.664	0.691	0.611	0.517	0.795	0.629	0.659	0.815	0.374	0.052	-0.287	1.000	-0.083	0.575
VEGF-A	-0.229	-0.117	-0.071	-0.016	0.303	0.484	-0.046	-0.046	-0.084	0.042	0.660	-0.313	-0.064	0.544	0.040	0.175	-0.117	0.047	0.083	0.107	-0.040	0.015	-0.149	0.010	0.562	0.263	-0.083	1.000	0.112
IL-18	-0.181	-0.299	-0.107	0.110	-0.138	0.154	0.365	0.393	0.328	0.392	0.317	-0.281	0.367	0.236	0.609	0.563	0.175	0.597	0.320	0.500	0.537	0.346	0.370	0.204	0.194	0.067	0.575	0.112	1.000

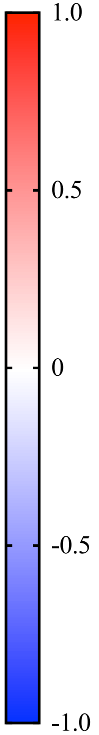

STROBE Statement - checklist of items that should be included in reports of observational studies

	Item No	Recommendation	Page No
**Title and abstract**	1	(*a*) Indicate the study's design with a commonly used term in the title or the abstract	1
		(*b*) Provide in the abstract an informative and balanced summary of what was done and what was found	1
**Introduction**			
Background/rationale	2	Explain the scientific background and rationale for the investigation being reported	2
Objectives	3	State specific objectives, including any prespecified hypotheses	2
**Methods**			
Study design	4	Present key elements of study design early in the paper	2, 3
Setting	5	Describe the setting, locations, and relevant dates, including periods of recruitment, exposure, follow-up, and data collection	2, 3, 4
Participants	6	~~(*a*) *Cohort study*—Give the eligibility criteria, and the sources and methods of selection of participants. Describe methods of follow-up~~ (n.A)	

		Case-control study—Give the eligibility criteria, and the sources and methods of case ascertainment and control selection. Give the rationale for the choice of cases and controls<br><br>~~*Cross-sectional study*—Give the eligibility criteria, and the sources and methods of selection of participants~~ n.A.	2, 3, 4
		~~(*b*) *Cohort study*—For matched studies, give matching criteria and number of exposed and unexposed~~ n.A.<br><br>***Case-control study***—For matched studies, give matching criteria and the number of controls per case	4
Variables	7	Clearly define all outcomes, exposures, predictors, potential confounders, and effect modifiers. Give diagnostic criteria, if applicable	3, 4
Data sources/measurement	8*	For each variable of interest, give sources of data and details of methods of assessment (measurement). Describe comparability of assessment methods if there is more than one group	3, 4
Bias	9	Describe any efforts to address potential sources of bias	4, 5
Study size	10	Explain how the study size was arrived at	3, Fig. 1

Quantitative variables	11	Explain how quantitative variables were handled in the analyses. If applicable, describe which groupings were chosen and why	4, 7, 8
Statistical methods	12	(*a*) Describe all statistical methods, including those used to control for confounding	4, 7, 8
		(*b*) Describe any methods used to examine subgroups and interactions	4, 7, 8
		(*c*) Explain how missing data were addressed	4
		~~(*d*) *Cohort study*—If applicable, explain how loss to follow-up was addressed~~ n.A.<br><br>***Case-control study***—If applicable, explain how matching of cases and controls was addressed<br><br>~~*Cross-sectional study*—If applicable, describe analytical methods taking account of sampling strategy~~ n.A.	N/A
		(*e*) Describe any sensitivity analyses	N/A

Results

Participants	13*	(a) Report numbers of individuals at each stage of study—eg numbers potentially eligible, examined for eligibility, confirmed eligible, included in the study, completing follow-up, and analysed	3, Fig. 1
		(b) Give reasons for non-participation at each stage	3, Fig. 1

		(c) Consider use of a flow diagram	Fig. 1
Descriptive data	14*	(a) Give characteristics of study participants (eg demographic, clinical, social) and information on exposures and potential confounders	5, Table 1
		(b) Indicate number of participants with missing data for each variable of interest	5, Table 1
		~~(c) *Cohort study*—Summarise follow-up time (eg, average and total amount)~~ n.A.	
Outcome data	15*	~~*Cohort study*—Report numbers of outcome events or summary measures over time~~ n.A.	
		Case-control study—Report numbers in each exposure category, or summary measures of exposure	5, 6, 7, STable 3, STable 4
		~~*Cross-sectional study*—Report numbers of outcome events or summary measures~~ n.A.	
Main results	16	(*a*) Give unadjusted estimates and, if applicable, confounder-adjusted estimates and their precision (eg, 95% confidence interval). Make clear which confounders were adjusted for and why they were included	5, Table 2
		(*b*) Report category boundaries when continuous variables were categorized	N/A
		(*c*) If relevant, consider translating estimates of relative risk into absolute risk for a meaningful time period	N/A

Other analyses	17	Report other analyses done—eg analyses of subgroups and interactions, and sensitivity analyses	7, 8, Fig. 3, SFig. 1, SFig. 2
**Discussion**			
Key results	18	Summarise key results with reference to study objectives	8
Limitations	19	Discuss limitations of the study, taking into account sources of potential bias or imprecision. Discuss both direction and magnitude of any potential bias	11
Interpretation	20	Give a cautious overall interpretation of results considering objectives, limitations, multiplicity of analyses, results from similar studies, and other relevant evidence	8-11
Generalisability	21	Discuss the generalisability (external validity) of the study results	8-11
**Other information**			
Funding	22	Give the source of funding and the role of the funders for the present study and, if applicable, for the original study on which the present article is based	12

*Give information separately for cases and controls in case-control studies.
